# Microbial Colonization and Inflammation as Potential Contributors to the Lack of Therapeutic Success in Oral Squamous Cell Carcinoma

**DOI:** 10.3389/froh.2021.739499

**Published:** 2021-10-04

**Authors:** Zoya Kurago, Jenni Loveless

**Affiliations:** ^1^Augusta University Dental College of Georgia, Augusta, GA, United States; ^2^Medical College of Georgia, Augusta, GA, United States; ^3^Georgia Cancer Center, Augusta, GA, United States

**Keywords:** microbiome, toll-like receptors, oral squamous cell carcinoma, oral epithelial dysplasia, inflammation, carcinogenesis, tumor microenvironment, oral mucosa

## Abstract

This review discusses the microenvironment of evolving and established conventional oral squamous cell carcinoma, by far the most common oral cancer. The focus of this paper is mainly on the more recent data that describe the role of microorganisms, host-microbial interactions, and in particular, the contributions of cell-surface toll-like receptors on immune system cells and on normal and malignant epithelial cells to their functions that support carcinogenesis. Because carcinomas arising at various host surfaces share much in common, additional information available from studies of other carcinomas is included in the discussion. Accumulating evidence reveals the complex toll-like receptor-mediated tumor-supporting input into many aspects of carcinogenesis via malignant cells, stromal immune cells and non-immune cells, complicating the search for effective treatments.

## Introduction

The vast majority of oral cancer cases are represented by squamous cell carcinoma (SCC) arising at the mucosal surface of the oral cavity. In distinction from oropharyngeal SCC, high-risk Human papillomavirus is rarely associated with oral SCC (OSCC), for which the known major risk factors are tobacco, alcohol, betel quid, especially when used together [1]. Additional risk factors are suspected, because a growing number of OSCC develop in the absence of the known risk factors. Several types of SCC are currently recognized, ranging from those only locally aggressive (verrucous carcinoma) to those capable of metastasis [2]. By far the most common and widely studied is the so-called conventional SCC, a somewhat heterogeneous group ranging in the level of differentiation and aggressiveness. Surgery, irradiation and platinum-based chemotherapy have been the typical treatment modalities for decades with rather little improvement in the impact on patient survival [3]. More recent developments include molecular agents that target important cancer pathways, as well as immunotherapy directed at boosting the adaptive immune response. While newer modalities show promise, particularly when used in combination, the improvements in prognosis so far are small, which necessitates further consideration of other mechanisms.

Another area of study that has the potential to significantly impact upon the approach to patient treatment is the complex host-microbiome interaction network operating throughout the natural history of carcinogenesis in the oral mucosa. Oral mucosa, whether normal or transformed, is colonized largely by commensal organisms, shifting in composition under changing conditions. The resulting interactions may affect both the host and the microbiome, likely adapting to, and influencing, the course of carcinogenesis. So far, no specific microorganism is known to initiate oral carcinogenesis, but evidence does support a role for host-microbial interactions in cancer progression, which is the focus of this review. These interactions will be discussed in the context of normal and transformed surface epithelium.

In this review we attempt to integrate current information on several aspects of oral squamous carcinogenesis, including the colonizing microorganisms, inflammation, and the functions of cell-surface innate pattern recognition receptors that initiate inflammatory and epithelial cell-intrinsic reactions, together capable of promoting carcinogenesis. At first, a discussion of normal structure-function will be presented in order to outline the relationship of the components of interest in the absence of malignant epithelial transformation for comparison with structure-function in carcinogenesis.

## Oral Mucosa: A Brief Overview

The oral cavity is the “lobby” of the digestive tract, a tube subdivided into segments with distinct functions, yet sharing a common goal and some environmental similarities. Under normal conditions, the mucosal surface of the digestive tract begins with stratified squamous epithelium of the oral cavity, pharynx, and esophagus, then changes to glandular epithelium in the stomach, small and large intestines, and back to stratified squamous epithelium at the anorectal junction, reflecting the distinct primary functions of the individual segments. The entire inner surface is continuously exposed to combinations of resident and transient substances and microorganisms, and the epithelial lining, with the help of associated cells and structures, performs barrier function. Areas covered by stratified squamous epithelium deal largely with mechanical forces required to move relatively coarse material and other friction-generating activities (e.g., speech). The tight multilayered structure of the stratified squamous epithelium overlying the basal proliferating cells is well-designed as a strong barrier, especially if the epithelium is keratinizing (forms a stratum corneum). Oral surfaces exposed to more mechanical stress because of mastication, such as gingiva, hard palate, and dorsal surface of the tongue, are covered by keratinizing epithelium, in contrast to non-keratinizing stratified squamous epithelium of the ventral tongue, floor of mouth, buccal, and labial mucosa [4]. The transition to glandular epithelium of the stomach and intestines is necessary for digestion and absorption, while still maintaining a selective barrier.

Besides epithelial cells, immune system dendritic Langerhans cells are normal residents of the stratified squamous epithelium. Additional specialized populations within oral epithelium include melanocytes, Merkel cells, and in some areas of the mouth, taste buds [4]. With the exception of taste buds, specialized populations generally localize in the deep layers, in proximity to the undifferentiated epithelial cells that replenish the rest of the compartment, although Langerhans cells may move up and extend their processes to the epithelial surface. The epithelial cell populations of the stomach and intestines are more variable, and non-epithelial cell types, including mucosa-associated lymphoid tissue (MALT), are found in some portions of the GI tract and the oropharynx. Because MALT is not a feature of the oral mucosa, it is outside the scope of this review. While the structure of the gastrointestinal (GI) tract surfaces is distinct, certain aspects of GI inflammation and carcinogenesis are relevant to the understanding oral carcinogenesis.

The mucosal lamina propria throughout the entire tract is separated from the epithelium by a basement membrane, and is designed to support the local mucosal functions. The typically loose fibrovascular connective tissue of the lamina propria contains both blood and lymphatic vessels, nerve bundles, resident dendritic cells, macrophages, and mast cells, as well as transient leukocyte populations. Salivary gland ducts traverse the lamina propria toward the epithelial surface in gland-bearing oral mucosal sites of the tongue, floor of mouth, labial and buccal mucosa, and posterior palate. Structures deep to the lamina propria differ from one subsite to another.

## Structural Landscape of Developing OSCC

OSCC develop most often at oral subsites that generally follow geographic variations in population exposures to carcinogens. In the USA, 60% of OSCC occur in the tongue and floor of mouth (sites most exposed to tobacco smoke and alcohol), while in Southeast Asia the most common area is buccal and labial mucosa (typical site for application of the potent carcinogen betel quid), and in Nigeria, 55% of OSCC develop in the gingiva and palate [5–7].

**Figure 1:**
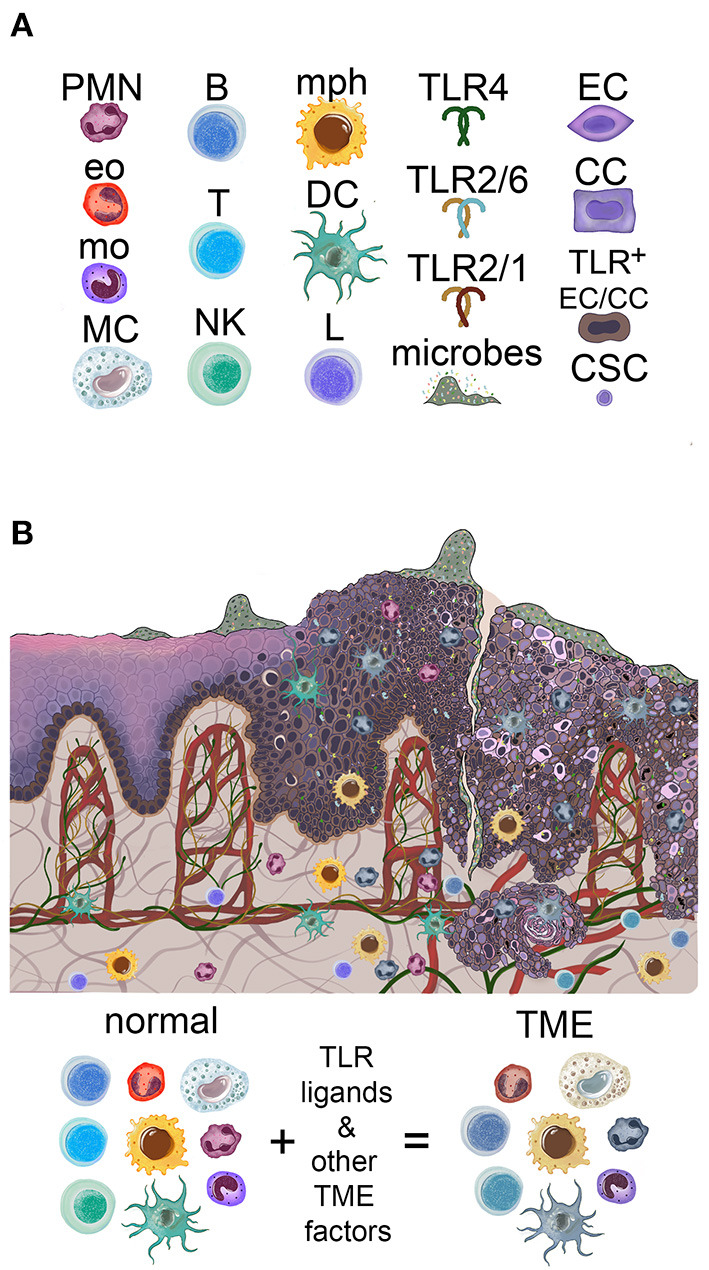
Overview of the oral mucosal environment in the evolution of oral squamous carcinogenesis. **(A)** Icons used to depict components of the figures in this review. PMN, neutrophil; Eo, eosinophil; mo, monocyte; MC, mast cell; B, B cell; T, T cell; NK, NK cell; mph, macrophage; DC, dendritic cell; L, lymphocyte; TLR, toll-like receptor; EC, epithelial cell; CC, cancer cell; CSC, cancer stem cell. **(B)** Oral mucosa is colonized by microbes, increasing in amount and penetration with carcinogenesis. Normal basal keratinocytes express high levels of TLR2, possibly both cell-surface and cytoplasmic, according to immunohistochemistry. High levels of TLR2 (as well as TLR5) are found in epithelial dysplasia keratinocytes and OSCC cells. The infiltrating immune system cells increase with progression to dysplasia and OSCC and acquire pro-tumor phenotypes in the TME of established OSCC.

There are several potentially malignant lesions in the oral mucosa [2]. A common early process in oral carcinogenesis that can progress to conventional SCC is the so-called pre-malignant lesion known as epithelial dysplasia (or intraepithelial neoplasia). Early in the process, as the stratified squamous epithelium undergoes transformation, immature atypical keratinocytes originating in the basal layer increasingly occupy more and more of the epithelial compartment, which is usually graded by pathologists as mild, moderate or severe dysplasia, or carcinoma-*in-situ*—a pre-invasive squamous carcinoma still restricted to the epithelial compartment by the basement membrane [1]. Another, simpler 2-tier system of grading early changes that includes all pre-invasive epithelial transformation is low-grade vs. high-grade dysplasia. Once the transformed epithelial cells breach the basement membrane and move into the lamina propria, the lesion is recognized as invasive squamous cell carcinoma. The loss of epithelial differentiation and maturation leads to structural abnormalities with functional implications. For example, the epithelium can become more permeable due to losses of cellular keratin and disrupted cell-cell adhesion and organization, as demonstrated in studies of skin epidermis [8, 9]. Skin and digestive tract studies indicate that outside-in permeability affects penetration of carcinogens, microbes and microbial products. Inside-out permeability has the potential to impact upon microbial colonization and activity, although the latter has not been tested. The reported changes in oral microbial colonization and virulence (discussed below) may also contribute to epithelial abnormalities and increasing mucosal inflammation. New blood vessels (angiogenesis, vasculogenesis) develop as a consequence of carcinogenesis and inflammation. The abnormal differentiation and uncontrolled growth contribute to disordered OSCC architecture and to the disruption of barrier function, in part through loss of surface continuity in the form of ulcers. SCC are usually ulcerated, which allows microbes and their products to access the connective tissue [10], fueling inflammation. OSCC progress by local invasion into adjacent tissues and metastasize first to regional lymph nodes, then to distant sites. The landscape of the mucosal environment in normal, precancerous, and OSCC conditions is depicted in Figure 1.

## Microbes

Studies of oral microbiome composition and its relationship to OSCC are reviewed extensively elsewhere, so only a brief overview is provided here. Commensal microorganisms, including eubacteria, archaea, protists, fungi, and viruses, inhabit all the epithelial barrier surfaces of the body, where bacteria, in particular, are as numerous as human cells [11, 12]. Oral mucosa is such a surface, and many different types of commensal bacteria appear to constitute the largest group of colonizers [11, 13–15]. Digestive tract segments – oral cavity, esophagus, stomach, small and large intestines—carry different and partially overlapping microbiomes [11], and the intraoral subsites also vary in the amount and diversity of associated organisms. For example, microbial composition of the dorsal tongue with its papillae is much more diverse than that of the buccal and palatal mucosae. In particular, both dorsal tongue and gingival crevices are home to aerobic and anaerobic species that form complex interactive communities, while smooth buccal and palatal mucosae are colonized by aerobes [16]. The normally symbiotic or tolerant relationships between the host and commensals contribute to barrier protection and are important for resistance to colonization by pathogens [17].

Microbiome studies related to oral carcinogenesis report various results. Human observational studies of microbial composition differ because of variations in populations, habits, presence of inflammatory oral disorders, as well as methods of sample collection and analysis [10]. For instance, key OSCC-associated risk factors alone differentially affect the oral microbiome, as revealed by analysis of oral rinses in American adults who are smokers [18] or consumers of alcohol [19]. Investigators also point out that organisms potentially important early in carcinogenesis may no longer be present or enriched in lesions at later stages. A recent study of Indian OSCC patients found distinct microbiomes on surfaces vs. deep intratumoral sites [10], and microorganisms have also been identified in lymph nodes with metastatic OSCC [20].

Although much remains unknown, two oral commensals and periodontal pathogens are under intense interrogation. G-negative anaerobe *Fusobacterium (F.) nucleatum* in particular has received significant attention in part because of its enrichment at surfaces of early pre-cancerous lesions [21] and at late stages [22] of oral carcinogenesis. One important virulence factor of *F. nucleatum* is the adhesion molecule FadA which inactivates E-cadherin and causes increased epithelial permeability, as well as facilitates beta-catenin- and WNT-mediated epithelial proliferation, at least demonstrated in colorectal cancer [23, 24]. *Porphyromonas (P.) gingivalis* is another anaerobic G-negative opportunist in the spotlight. Both *F. nucleatum* and *P. gingivalis* are often investigated together for potential roles in oral carcinogenesis because of their contributions to periodontal disease, collaborative growth, an association between periodontal disease and oral cancer [25], and enrichment in the microbiome of both colorectal adenocarcinoma and the pre-cancerous colorectal adenoma [15, 22, 26]. Notably, the majority of epithelial dysplasias do not progress to OSCC, and some subtypes of OSCC develop in the absence of preceding epithelial dysplasia. So far there have been no longitudinal microbiome studies of progressive vs. non-progressive epithelial dysplasia. Other potentially premalignant oral epithelial conditions have not been sufficiently characterized.

Despite study variations, there is overall agreement that the progression from normal to dysplastic squamous epithelium on to OSCC is accompanied by significant shifts in diversity and abundance of microorganisms localized to the lesions, as well as the oral microbiome in general. It is possible that cancer progression and shifting microbiome occur through coevolution, impacted by risk factors and a changing microenvironment.

To test the impact of the oral and tumor-associated microbiomes on OSCC development, several studies have utilized animal models, such as SCC induction by the tobacco-like carcinogen 4-nitroquinolone (4-NQO), or transfer of established human or murine OSCC cells into animals [15]. Similarly, both human and murine oral microbiomes have been evaluated. Overall so far, these studies show that the presence of either selected candidate organisms [26–28] or whole microbiome derived from established tumors, promoted cancer progression in colorectal cancer [29] and OSCC [30]. Even more interesting is that 4-NQO-treated mice colonized by oral microbes from healthy animals developed more and larger OSCC than germ-free 4-NQO-treated mice [30]. In other words, there is evidence that multiple distinct combinations of bacteria identified in either normal conditions or premalignant-malignant lesions are able to enhance the growth of squamous carcinomas initiated by chemical carcinogens. However, a limitation of the reported studies about OSCC growth in germ-free animals is that they have not examined the host immune cell populations and their functions in the associated mucosa. Given that germfree mice are deficient in bone marrow–derived peripheral myeloid populations [31], it is possible that lack of tumor growth in germ-free conditions was at least partly due to the lack of myeloid cells in the tumor microenvironment, disrupting myeloid cell-microbiome-tumor cell interactions.

The inconsistent data about the composition of the OSCC-associated microbiome may be partly balanced by the results of functional analyses of individual species and their combinations. For example, different microorganisms can live in similar conditions and produce similar metabolites [15]. Acetaldehydes, N-nitroso compounds, volatile sulfur compounds, organic acids, and other bacterial metabolites, are capable of causing DNA damage (genotoxicity) and are produced by various organisms found in normal and OSCC-associated microbiomes [15, 32]. Interesting recent advances in metatranscriptomics have revealed that despite variable composition, there was significantly higher microbial metabolic activity and expression of putative virulence factors in established OSCC, with *F. nucleatum* in the lead, relative to matched sites from tumor-free controls [33]. Increased presence and virulence of certain OSCC-associated bacteria may also be due to the loss of species that otherwise antagonize them [25, 34]. Notably, most of the published reports are about bacteria, in part because many other oral microbiome members have only been discovered recently, and many are not cultivable. The best studied fungal organisms often associated with OSCC are *Candida* spp. the biology of which is also capable of supporting carcinogenesis [35, 36]. Together, evidence indicates that the microbiomes of established OSCC, regardless of composition, are highly active, and that the products of their metabolism have the potential to directly affect host epithelial cells. It is reasonable to expect that the host-microbiome influences in the evolving OSCC are bidirectional: sharing nutrients, generating and processing metabolites, competing for critical materials and spaces.

A key point of interest for further discussion is that, while the environment of oral epithelial dysplasia and OSCC exhibits shifts in microbial abundance, diversity, and metabolic activities, current understanding is that the colonizers are a mix of commensals and some opportunistic pathogens, all expressing microbe-associated molecular patterns (MAMP) that are common to commensals and pathogens. MAMP are recognized by specific host pattern recognition receptors (PRR) in immune and non-immune cell types, triggering inflammation and other responses. PRR include toll-like receptors (TLR), C-type lectin-like receptors (CLR), and NOD-like receptors (NLR) [37]. Here we will focus on the best studied group, the TLR1-6 expressed on cell surfaces.

## PRR: Toll-Like Receptors (TLR)

TLR are type I transmembrane proteins with critical functions in cells of the immune system. Out of the ten human receptors in this family, the intracellular TLR3, 7, 8, and 9 are localized to acidic organelles called endosomes and recognize microbial nucleic acids, making this group of receptors dependent on phagocytic properties of cells and/or intracellular infections. In contrast, TLR1, 2, 4, 5, and 6 are more accessible and expressed on the plasma cell membranes, and as such detect distinct molecules of microbial surfaces, although TLR4 also associates with endosomes [38]. TLR10 is also expressed on the plasma cell membrane and may be mainly an inhibitory molecule, although the natural ligand for this receptor is unclear [39]. Mice have TLR1-13, the first nine of which are the best studied and are similar to human TLR1-9 in the specificities and functional activities, which is why mice are widely used for TLR-related studies relevant to humans. The cell-surface TLR are of special interest in the context of mucosal function and surface-associated carcinogenesis and will be discussed in more detail.

While exposure to their specific MAMP causes TLR to form homodimers, TLR2 also heterodimerizes with either TLR1 or TLR6, which generates broader recognition of MAMP. TLR1 and TLR6 are not known to function independently of TLR2. TLR2/1 dimers bind bacterial triacylated lipopeptides/lipoproteins, and TLR2/6 dimers interact with diacylated forms of these molecules [40]. Lipoproteins are components of cell walls of G-positive and G-negative bacteria [41]. Both forms of lipoproteins may be expressed in the same species, depending upon the environmental conditions—pH, growth phase, temperature, and salt concentration [40, 42]. Furthermore, peptidoglycan and lipoteichoic acid, thought to interact with TLR2, may not be true TLR2 agonists according to investigations that used more stringent purification methods and other approaches [40]. Molecules and receptors other than TLR2 have been shown to bind peptidoglycans [43]. TLR2/6 also binds fungal cell wall zymosan, a protein-carbohydrate complex. In contrast, TLR4 recognizes the glycolipid lipopolysaccharide (LPS) of most G-negative bacteria. TLR2 and TLR4 are designed to accept transfer of ligands from other host molecules. Molecules that collaborate with TLR2 are CD14, mannose-binding lectin, CD36, and Dectin-1, while TLR4 works with LPS-binding protein (LBP), CD14, and MD2 [38]. The utility of these collaborators is especially evident in responses to whole bacteria. Moreover, several endogenous molecules collectively called danger-associated molecular patterns (DAMP) are reported to also trigger TLR2 or TLR4 activation [44]. Most known DAMP appear to activate TLR indirectly by complexing with other TLR-binding molecules [45]. Of special interest are two DAMP that have TLR-binding and activation abilities, and they are increased during inflammation and cell death, which are abundant in cancer. The high mobility group protein 1 (HMGB1) is fully confirmed to bind TLR4 and trigger its dimerization and signaling without interfering with the LPS-binging site [46], while versican is reported to bind and activate the TLR2/6-CD14 complex [45, 47]. Finally, TLR5 is specific for flagellin, the principal component of flagella in motile organisms, which is often found in pathogens (ex. *Salmonella* and *Helicobacter pylori*) and contributes to their virulence [38, 48].

Either commensal or pathogen MAMP binding induces TLR dimerization and signaling. The outcome of signaling depends upon the specific TLR, the cell type, and cell state. Innate immune system monocytes, macrophages and dendritic cells (DC) express high levels of all or most TLR, depending upon cell subset, and have yielded most of the accumulated information about TLR function. TLR signaling is detailed in a recent review by Fitzgerald and Kagan [38] and Vijayan et al. [48]. In brief, ligand-induced activation of all TLR, except TLR3, triggers the assembly of the intracellular multimolecular complex called the myddosome (named after myeloid differentiation primary response protein, or MyD88), which then activates several pathways: canonical NF-kB, mitogen-activated protein kinase (MAPK), and change in metabolism (induction of glycolysis). The endosomal TLR3 activity utilizes the signaling complex called triffosome (named after TIR domain-containing adaptor inducing interferon-β, or TRIF), inducing canonical NF-kB, interferon regulatory factor 3 (IRF3), and other less well-characterized activities. TLR4 is unique in that it uses both the myddosome and the triffosome. Interferon-alpha (IFN-alpha) production is triggered via the triffosome, although in some cells and in response to certain ligands, IFN-alpha may also be induced via the myddosome. The inflammatory pathway signaling in macrophages and DC is further amplified by TLR-induced cytokines that also activate NF-kB (especially IL-1 and TNF-alpha) [38, 48]. Because excessive TLR activity is dangerous to the host [49], there are various levels of cell-intrinsic and extrinsic negative regulation of TLR activities [50, 51]. How negative regulation works in carcinogenesis is under investigation.

## TLR Expression and Function at Mucosal Surfaces

Symbiosis with, and tolerance to commensals is the normal state of affairs at surfaces, but this relationship depends upon continuous MAMP recognition via PRR on epithelial and innate immune system cells, the combined efforts of which, in addition to secreted soluble factors (antimicrobial peptides, IgA, other), limit surface penetration by commensal and pathogenic microorganisms [17]. Epithelial MyD88-dependent TLR are required for homeostasis, which was demonstrated in the gut epithelium [52], in epidermal keratinocytes [53] and other cell types, at least in part through induction of cytoprotective factors IL-6, KC-1, and other molecules. Remarkably, signals from the gut microbiome through host PRR have impact well-beyond the gut. These signals control the production, migration and functions of host innate and adaptive immune system cells systemically, which in turn, affects the ability of the host immune system to fight pathogenic infections, as well as local and remote cancer growth and treatment [31, 54–57]. While TLR are expressed by many cell types, the following discussion is focused on immune system cells and epithelial cells because of their critical roles in the mucosa.

### Cell-Surface TLR in Mucosal Immune System Cells

As normal residents at mucosal surfaces and professional immune system cells, DC and macrophages express high levels of cell-surface and intracellular TLR and are critical for defense against pathogens, but are also important for controlling commensals. Intact barrier generally prevents TLR activation on DC and macrophages, and antigens captured by DC and presented to T cells in such homeostatic conditions lead to tolerance. In contrast, if DC acquire antigens during infection or inflammation, co-stimulatory molecules are expressed, and these DC induce long-term T cell activation [58]. DC with activated PRR readily migrate from infected or otherwise damaged and inflamed tissue to the draining lymph nodes where naive T cells are activated and in turn, contribute to inflammation in the mucosa. Other resident cells common throughout the mucosal tissues are known as innate lymphoid cells (ILC), which contribute cytokines that affect the type of adaptive response, although the role of TLR in these responses is mostly unknown [59].

The type of adaptive response depends on additional factors, including cytokines, and the specific TLR activity affects the profile of the induced cytokines [60]. The patterns of responses are typically classified as Th1 (type 1), Th2 (type 2), and Th17 (type 17) with additional subtypes. Although T helper (Th) cell subsets are not the only population involved, “Th” rather than “type” will be used in this review to avoid confusion with the hypersensitivity reactions, which are classified as Types 1–4. In general, Th1 responses include IFN-gamma-producing Th1 and T cytotoxic cells, desirable against intracellular pathogens and cancer cells, and require IL-12 and IL-18 induced in DC and macrophages via TLR-MyD88-dependent pathway [61–63]. TLR4 and TLR5 signals contribute to Th1 bias as they induce DC to make IL-12. Macrophages and DC that make these IFN-gamma-inducing factors are known as M1 and DC1, respectively. In the context of an infection, IFN-gamma stimulates macrophage activity, including increase in TLR expression and activity [64]. Th2 responses are initiated by tissue-resident innate lymphoid cells 2 (ILC2) but also require macrophages, DC and Th cells. Macrophages and DC that support type 2 responses are known as M2 and DC2, respectively. TLR2 signals contribute to a Th2 bias, as IL-12 is not induced. Th2 reactions are characterized by IL-4, IL-5, IL-9, and IL-13 cytokines, and are important in responses to parasites, allergens, an array of microbial pathogens and endogenous molecules [37, 65]. On the other hand, Th17 responses are characteristic of many infections by extracellular bacteria and fungi as well as non-infectious chronic inflammatory disorders. Depending upon the specific conditions, Th17 responses develop when several possible combinations of IL-1beta, TGF-beta, IL-6, and IL-23 are present. Besides inducing Th17 cells, IL-23 activates other T cells, and innate immune system cells that are also important for Th17 responses [66]. Earlier studies showed that TLR4 and TLR5 signaling could mediate a Th1 bias, while TLR2 ligation produced a Th2 bias [60]. Recent evidence suggests that TLR2 activity drives Th17 inflammatory gene expression and may be sufficient for CD4^+^ T cell-mediated autoimmune reactions [67]. Th17 factors were also induced in response to TLR5 activity in innate lymphoid cells and DC [68].

Macrophages with their high levels of TLR are critical mucosal lamina propria responders to MAMP near surfaces. It turns out that these responses are fine-tuned early on, to avoid premature inflammatory reactions related to TNF-alpha toxicity and unnecessary disruption of mucosal homeostasis. Studies using TLR4 and LPS showed that the two pathways downstream of the receptor, NF-kB and MAPK, have different thresholds for activation: low doses of LPS seen in homeostatic conditions only activate NF-kB without triggering MAPK, while both pathways are required to produce inflammatory mediators [69]. This is important in view of ubiquitous presence of TLR ligands in the periphery in the absence of overt infections or tissue damage. The described mechanism is only one of many ways to negatively regulate TLR signaling and avoid unnecessary inflammation. Whether other cell surface TLR follow the same rules has not been established.

TLR signals can also directly modulate the activities of the adaptive immune system B cells and T cells, which are numerous in MALT and at sites of inflammation, but much more sparse in the oral mucosa under normal conditions. B cell antibody profiles vary depending upon the type of response and whether T cell help is required, i.e. are T cell-dependent vs. T cell-independent. B cells integrate signals from their own TLR and the B cell receptor (BCR), which directs antibody production and interactions with T cells. Moreover, TLR activity in B cells stimulates their proliferation [70]. TLR2/1 activity can rescue chronically activated “exhausted” Th1 cells by modulating immune checkpoint molecules, as well as promote the function of cytotoxic T cells, while also assisting the differentiation and function of Th17 cells [71]. On the other hand, TLR2 activation along with other factors, promotes regulatory T cells (Treg) that are important for shutting down destructive inflammatory reactions. Treg cells and DC were shown to maintain gut-microbiota homeostasis and mitigate inflammation and microbial pathogenicity in part because they activate B-cells to release secretory IgA for specific and non-specific coating of surface microbes, thus preventing their direct contact with the epithelial surface. This process is directed in the presence of cytokines IL-4, TGF-β, IL-5, IL-6, and IL-10 [14, 72, 73].

Notably, the oral mucosal subsites vary in normal immune cell composition. Gingiva with its thinner epithelium in the gingival sulcus than any other oral mucosal surface, has the privilege of continuous exposure to dental plaque bacteria. Under normal conditions, this subsite was found to contain neutrophils, antigen presenting cells, ILC, and resident memory T cells (many more CD4^+^ than CD8^+^). The most significant distinction of the gingival sulcus mucosa from buccal mucosa was the high numbers of neutrophils [74], suggesting that MAMP penetration past the sulcular epithelial barrier occurs there more easily. Even in the absence of recognizable clinical signs of inflammation, IFN-gamma and IL-17 were the dominant cytokines identified in the sulcular mucosa in this study, consistent with Th1 and Th17 responses. The numbers of CD4^+^ T cells, neutrophils and B cells increased significantly in periodontitis, a common chronic inflammatory condition that involves the gingiva, periodontium, and underlying bone. In other studies, IL-17 produced by Th17 cells was the primary soluble factor detected in periodontitis [75, 76].

In general, current evidence indicates that oral mucosal immune cell responses to TLR ligands under normal conditions depend first and foremost on normal resident DC and macrophages and the penetration of TLR ligands past the epithelial barrier, the extent of which varies between subsites. The epithelial cells develop their own response pattern discussed below.

### Epithelial Cell-Surface TLR

Cell surface TLR1-6 are present and functional in skin and mucosal epithelial cells with some variation in the exact distribution of individual receptors and their functions in homeostatic vs. inflammatory settings. For example, murine digestive tract epithelial TLR2, TLR4, and TLR5 were found at different levels in segments of the small and large intestines [77]. The level of epithelial cell responsiveness is determined by the levels of TLR expression [77], and the type of response may also depend upon the state of epithelial cell differentiation.

Remarkably, multiple studies reveal that TLR2 with its partners TLR1 and TLR6 is particularly important for barrier function in the epidermis and mucosal epithelia. TLR2 activity enhances tight junctions in the epidermis [78, 79], augments squamous epithelial barrier in the esophagus [80], and is upregulated in intestinal epithelial cells where it stimulates epithelial turnover in response to microbial colonization [81], as well as reduces permeability and mucosal inflammation by preserving tight and gap junctions in the intestinal epithelium [82, 83]. On the other hand, the levels of TLR2 and TLR4 increase markedly in epidermal keratinocytes exposed to IFN-gamma and LPS [84], and Th2 inflammation also contributes to increased permeability of the epidermis and airway epithelium [85]. Similarly, IFN-gamma and TNF-alpha were shown to disrupt intestinal epithelial permeability via effects on tight junctions [86].

Both TLR2 and TLR4 are reported to contribute to functions of epithelial stem cells which are capable of mitosis. Epidermal keratinocyte TLR2 contributes to skin wound healing by stimulating undifferentiated keratinocyte proliferation and migration, and in this manner, it promotes barrier recovery [87, 88]. TLR2^−/−^ or MyD88^−/−^ mice exhibit compromised repair of intestinal surfaces, because TLR2/MyD88 pathways are important for stimulating intestinal epithelial stem cell pool expansion after injury [89]. On the other hand, TLR4 activity inhibits intestinal stem cell proliferation and promotes apoptosis [90].

Although only minimal information on oral epithelial subsite TLR1-6 expression and function is available, there is evidence that both may depend upon the presence of mucosal inflammation, similar to observations in the epidermis. In the absence of inflammation, oral epithelial TLR2 and its' partners are expressed in human and murine specimens as well as in purified epithelial cells. Immunohistochemical evaluation of human oral mucosal specimens revealed that in normal epithelium, TLR2 was clearly expressed in the basal and parabasal keratinocytes, while few differentiated spinous layer keratinocytes showed rare perinuclear granular staining [91]. *In vitro*, normal oral keratinocytes are more likely to respond to TLR2/1 and TLR2/6 agonists than to TLR4 stimuli [92–98]. These responses include the production of antimicrobial peptides and under some conditions, relatively small amounts of cytokines and chemokines. Similar to epidermal, esophageal, and intestinal epithelia, gingival keratinocytes upregulated tight junction proteins in response to the TLR2-specific *P. gingivalis* or its purified LPS, which decreased barrier permeability *in vitro* [94].

Notably, TLR expression and responsiveness in oral keratinocytes in reactive conditions is reported to increase. Inflamed gingival epithelium in periodontitis expressed more TLR2 than normal gingival keratinocytes [93]. Similarly, keratinocytes in inflamed oral mucosa and in orthokeratosis—a common reaction to friction—showed diffuse TLR2 expression throughout the epithelial layers [91], indicating a positive correlation between inflammatory factors and increased epithelial TLR2 expression. *In vitro*, oral epithelial cells pre-exposed to IFN-gamma or IL-17, produced inflammatory mediators IL-1beta, TNF-alpha, and IL-8 (CXCL8) in response to TLR2 or TLR5 agonists [93, 99], suggesting that inflammatory “priming” may be a factor in order to recruit oral keratinocytes into the inflammatory process with subsequent increase in epithelial permeability [86].

As mentioned earlier, normal oral epithelial cell TLR4 expression and responsiveness appear to be limited [95, 100–102]. Gingival epithelial cells were shown to express intracellular endosomal TLR4, so that LPS bound and activated TLR4 after internalization [101]. Other primary oral keratinocytes are reported to produce antimicrobial peptides, but not cytokines or chemokines, when stimulated with LPS *in vitro* [97].

Few studies have so far evaluated TLR5 expression and function in the oral cavity. In the tongue epithelium, TLR5 was found in the basal and parabasal layers, increasing in superficial layers under inflammatory conditions [93, 102, 103] and replicating the pattern of TLR2 expression. Remarkably similar to observations in premalignant lesions of the stomach and esophagus [104, 105], keratinocytes in oral epithelial dysplasia showed strong cytoplasmic expression of TLR2 [91] and TLR5 [103].

**Figure 2:**
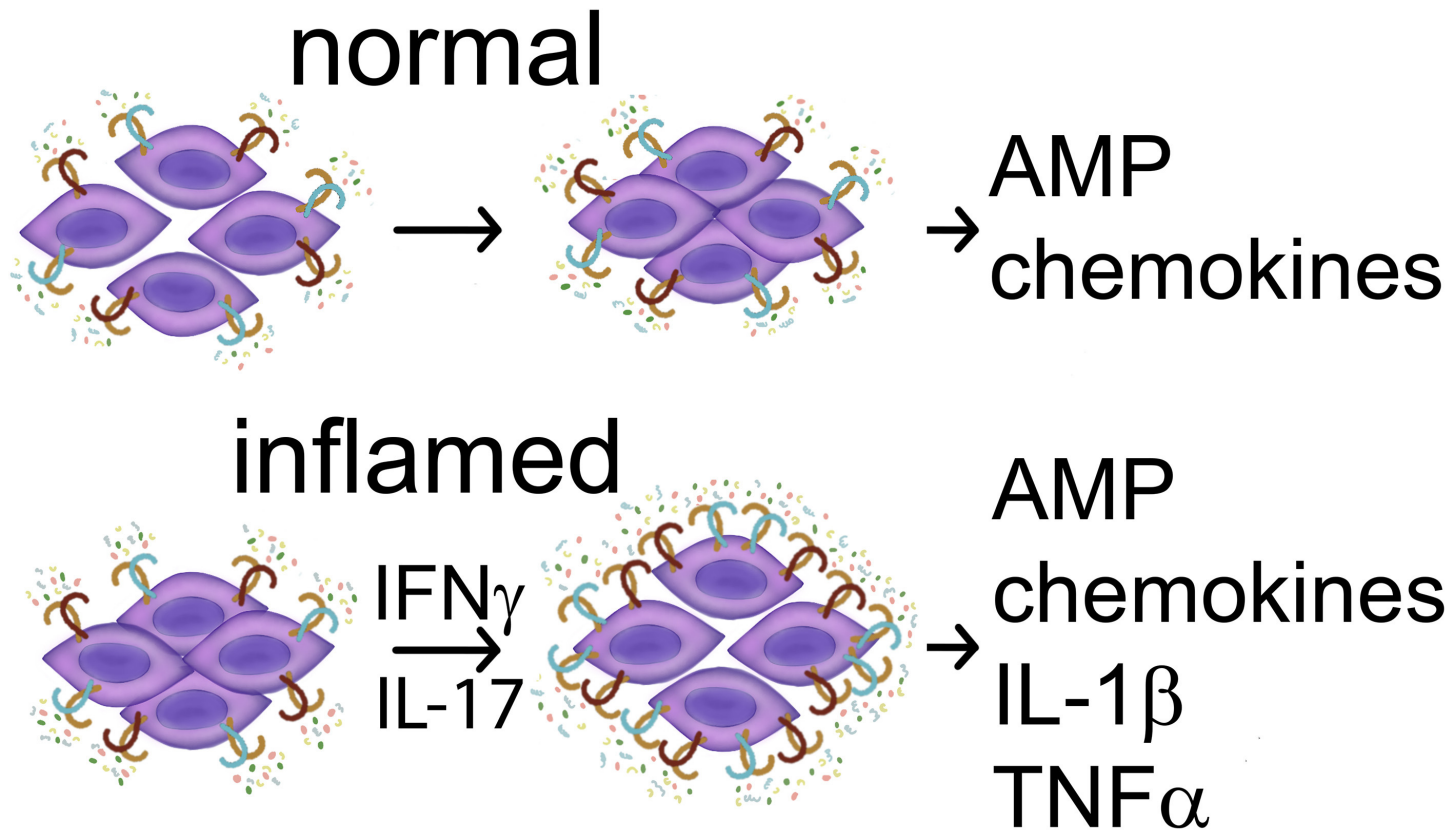
Summary of TLR activities in normal keratinocytes. **(Top)** In the absence of inflammatory cytokines TLR2 ligands stimulate a stronger barrier—reduced permeability and production of antimicrobial peptides (AMP), and may also induce chemokine CXCL8. **(Bottom)** In the presence of inflammatory mediators IFN-gamma or IL-17, TLR expression increases, and TLR ligands stimulate keratinocyte participation in inflammation with production of IL-1beta and TNF-alpha. TNF-alpha and possibly other inflammatory mediators contribute to increased permeability. References: Beklen et al. [92], Beklen et al., [93], Guo et al. [94], McClure and Massari [95], Mullin and Snock [86], Sugawara et al. [97], and Uehara et al. [99].

A brief summary of surface TLR in normal and abnormal oral squamous cells is provided in Table 1. Together, the limited available data suggest that the expression and function of cell surface TLR1-6 in the oral epithelium appears to be consistent with that at other epithelial surfaces, assisting homeostatic epithelial barrier function via tight junctions (TLR2 with partners) and induction of antimicrobial peptides, but increasing in expression and pro-inflammatory function under inflammatory conditions with increase in permeability (Figure 2). A brief summary of cell-surface TLR expression and function in normal, precancerous and malignant epithelial cells is provided in Table 1. It is possible that the limited epithelial responses to TLR stimuli are due to low levels of TLR expression under normal conditions. Much remains to be uncovered regarding oral subsite epithelial TLR performance in normal vs. pathologic conditions.

**Table 1:** Expression and functions of cell-surface TLR in normal and abnormal oral epithelium.

	**Expression**	**Reported responses**	**References**
Normal oral epithelium	TLR1-6; subsite variation; TLR2 and TLR5 mainly in basal layer	TLR2: AMP, chemokines; enhanced tight junctions/reduced permeability; TLR2>TLR4; TLR4 responses limited; may require internalization of LPS	[91–95, 99–103]
Inflamed oral epithelium or pre-exposed to IFN-γ, IL-17	Increased TLR2, TLR4, TLR5; others not tested	Production of IL-1-beta, TNF-alpha; increased epithelial permeability	[84, 91, 93, 94, 99, 102]
Oral hyperkeratosis	Increased TLR2	Unknown	[91]
Epithelial stem cells	TLR2, TLR5; others not tested	Not described in normal oral epithelial stem cells; promotes proliferation and migration in epidermal and intestinal epithelial stem cells	*Epidermal, intestinal:* Neal et al. [90], Schauber et al. [87], Schauber and Gallo [88], and Scheeren et al. [89]
Oral epithelial dysplasia	Increased expression in TLR2, TLR4, TLR5; others not tested	Unknown	[91, 93, 98, 103, 106]
PVL^*^	Increased TLR2	Unknown	[91]
OSCC	TLR1-6; increased TLR2, TLR5, (increased TLR4?)	TLR2-high OSCC: Induction of NF-kB, ERK1/2 MAPK; TLR2, TLR4: Induction of IL-6, GM-CSF, IL-1, TNF-alpha, CCL2, CCL20, CXCL8; VEGF	[28, 107–110]

*For more details please see text*.

* *PVL, proliferative verrucous leukoplakia*.

### Mechanisms of Mucosal Inflammation Shutdown

Timely shutdown of mucosal inflammation is important in order to repair damage and re-establish barrier. TLR signals trigger both destructive and restorative mechanisms that allow transition from pro- to anti-inflammatory process, and these are executed via multiple mechanisms, as illustrated in the following examples. Mucosal resident macrophages exposed to significant doses of TLR ligands quickly secrete TNF-alpha and IL-6, among other factors. On one hand, NF-kB-activating pro-inflammatory cytokine TNF-alpha promotes M1 macrophage phenotype important for infection control, actively preventing macrophages from becoming M2 [111]. On the other hand, IL-6 is known for its important cytoprotective role in tissue homeostasis by directing the survival, proliferation, and differentiation of immune system and epithelial cells through IL-6 receptors and signal transducer and activator of transcription (STAT)3 [112–114]. STAT3 also activates the production of vascular endothelial growth factor (VEGF) with subsequent angiogenesis required for tissue perfusion, infection control and then, healing. In addition, TLR agonists induce DC and macrophages to secrete IL-10 [115], which activates STAT3 in immune system cells. IL-10 is broadly anti-inflammatory, and is also secreted by other innate and adaptive immune system cells. Together, IL-6- and IL-10-mediated STAT3 activity contributes to negative regulation of the Th1 immune response in M1 macrophages and other involved cells. Moreover, TLR2 agonists among other factors induce Treg which secrete IL-10 and TGF-beta [115] with anti-Th1 input. TLR2 activity in monocytes not only induces M2, but also directs their differentiation into the monocytic myeloid-derived suppressor cells (M-MDSC), another important subset that inhibits Th1 T cells, cytotoxic T cells, NK cells, and DC. Even B cell TLR activity can trigger their regulatory function that suppresses Th1 and Th17 cells [116]. Transition to immunosuppressive and immunoregulatory profile is just as important for the epithelial cells around wounds and ulcers, as it permits epithelial proliferation and migration necessary to close the wound surface and repair the barrier; TLR2 stimuli facilitate this process in the skin [87]. Similarly, commensal colonization was shown to promote restoration of a functional physiological barrier in the intestines [117] which implicates TLR. Once again, IL-6 released in response to TLR activity is important for survival and proliferation of intestinal epithelium [112].

Another fundamental mechanism in the anti-inflammatory process and tissue repair is TLR-induced metabolic reprogramming of macrophages and purinergic signaling [118]. TLR activation in M1 macrophages leads to aerobic glycolysis with accumulation of lactate and production of inflammatory mediators and high levels of ATP. ATP released outside the cells is processed by cell surface CD39 and CD73, membrane-bound ecto-enzymes expressed by Treg and macrophages. These enzymes sequentially dephosphorylate ATP to adenosine, which then acts on inhibitory adenosine receptors (AR) A2a and A2b that are rapidly upregulated in macrophages in response to TLR activity. A2a and A2b activities then suppress the production of TNF-alpha and IL-12 [119–122], a cytokine necessary for T cell IFN-gamma production [61]. This is beneficial for the M1 to M2 switch and also because excess TNF-alpha is toxic to many cells [123] including monocytes [124], macrophages [125], endothelial cells [126], and cancer cells [127]. Moreover, adenosine inhibits NK cell and CD8^+^ T cell cytotoxicity, as well as promotes suppressive activity by facilitating expansion of MDSC and Treg [128]. The important role of A2a AR has been specifically demonstrated in wound healing [129]. The transition from M1 to M2 macrophage phenotype not only helps the immunosuppressive and immunoregulatory properties, but also activates other macrophage genes involved in tissue repair and resolution [130].

In summary, TLR at host surfaces trigger both destructive pro-inflammatory as well as reparative and anti-inflammatory mechanisms, the sequence and timing of which are critical in order to first control pathogen invasion, remove dead cells, and then to facilitate a return to homeostasis. All of these activities are observed, and often exaggerated, in the microenvironment of surface-associated carcinomas, thanks in part to unresolved surface breach that continues to fuel TLR activation in present and incoming cells.

## Cell-Surface TLR and Inflammation in the Pre-Cancer and Tumor Microenvironment (TME)

In this section, the main focus is on the impact of cell-surface TLR in immune system and epithelial cells on the biology of evolving OSCC with additional information from other surface pre-cancers and carcinomas. There is clear evidence that TLR activity triggered by MAMP or DAMP is highly relevant to the biology of carcinomas because of direct effects on TLR pathways and the consequences that arise from TLR-induced production of soluble factors. Because TLR activity and inflammation are tightly intertwined, they are discussed together. TLR initiate a cascade that begins with activation of canonical (classical) NF-kB and ERK MAPK pathways, amplified by induced host mediators, with subsequent enrollment of STAT3 activities, which affects essentially all cell types present in the tumor. NF-kB and STAT3 activities go hand-in hand in TME. The typical targets of classical NF-kB signaling include factors that affect many aspects of inflammation, cell recruitment, cell proliferation, survival or death, and angiogenesis, such as TNF, IL-1, IL-6, GM-CSF, CXCL8 (IL-8), CXCL1, CXCL2, CCL2, CCL3, CCL5, MMPs, cyclin D1, MYC, BCL-Xl, BCL2, FLIP, COX2, iNOS, VEGF [114], and the hypoxia-inducible factor (HIF)-1, a critical factor in cellular response to hypoxia [131]. On the other hand, the ERK MAPK pathway is responsible for basic cellular processes, including cell proliferation and differentiation, which are crucial for cancer cells, including cell proliferation, survival, growth, metabolism, migration, and differentiation [132]. The MAPK pathway is important for cell-intrinsic effects of TLR signaling.

### TLR and Pre-cancer

Limited information on TLR and inflammation in epithelial premalignant conditions is available. An inflammatory environment is not unique to established carcinomas, but is also a feature of their precursors. Given the disordered differentiation, loss of keratin expression [133] and frequent disruption of cell-cell adhesion in epithelial dysplasia, barrier function may be compromised so that MAMP could penetrate the barrier, activate macrophages, as well as stimulate DC maturation and migration to lymph nodes followed by activation of the adaptive response. The barrier permeability may be exacerbated by inflammatory factors, such as TNF-alpha, IFN-gamma, or IL-17, allowing more influx of microbial TLR ligands into the connective tissue. Moreover, DAMP released because of inflammatory host cell damage may also activate TLR.

Whether inflammation precedes and contributes to the initiation of oral carcinogenesis or not, premalignant lesions in the oral cavity, metaplasia and dysplasia in the esophagus and stomach, and adenoma in the colon—are typically associated with mucosal inflammation, while colitis-associated intestinal carcinogenesis is well-recognized. The epithelial cells in premalignant lesions of the oral, esophageal, gastric, and colonic mucosa express high levels of TLR2, TLR4, and TLR5 [91, 93, 98, 103–106, 134, 135], which could potentially contribute to the induction and persistence of certain aspects of inflammation in the mucosa in the context of compromised epithelial differentiation. Gastric carcinogenesis is associated with decreasing levels of TLR inhibitors and elevated TLR levels throughout the process of carcinogenesis starting with metaplasia through established adenocarcinoma [105]. Toll-like receptor 5 has also been proposed as a biomarker for gastric and cervical dysplasias, because its expression increases through stages of cancer development [105, 136]. However, the function of epithelial TLR in pre-cancer has been addressed only in a few studies of gastric and colon dysplasia. For example, in a mouse model of colon cancer, epithelial TLR4 activity induced the beta-catenin pathway, potentially linking TLR4 with oncogenesis [137]. How epithelial cell TLR function in oral premalignant lesions is unknown.

The inflammatory milieu associated with mucosal pre-cancer has been addressed in several studies. Colorectal adenomas are associated with Th17 responses, followed by immunosuppression in adenocarcinoma [138], and IL-17-related profile has been linked to carcinogenesis in the GI tract [139]. Similarly, inflammatory cell infiltrates and soluble factors found in the mucosa in oral epithelial dysplasia preferentially showed a Th17 profile [140, 141], although any specific contribution of Th17-related immune response to oral carcinogenesis is yet to be identified. A 4-NQO carcinogen-driven mouse model of oral cancer revealed that the initial inflammatory profile present in pre-cancer (dominated by IFN-gamma and IL-17 along with other inflammatory mediators) [140] was succeeded by anti-inflammatory cytokine IL-10 as the lesions progressed to SCC [142]. In a molecular analysis of human oral premalignant lesions (OPL) which included hyperplasia and dysplasia, samples were grouped into “immunological” and “classical” categories because of significant differences in the enrichment of immune pathways vs. xenobiotic metabolism pathways, respectively [143]. Although a detailed assessment of immune landscape was not described in this study, the “immunological” subset had more inflammatory cells within the epithelium and lamina propria, as well as an apparent enrichment of genes for cytotoxic T cell, IL-12, and IL-17 pathways, consistent with Th1 and Th17 profiles [143]. Exactly how TLR activities in immune and epithelial cells of oral premalignant conditions affect the milieu is yet to be characterized.

In order to understand if and how pre-existing oral mucosal inflammation of different types and its causes affect oral carcinogenesis, better models associated with inflammation are needed. In addition, studies of potentially malignant disorders separated by subsite, the presence of dysplasia, or inflammation are important.

### Established Cancer TME

#### Overview of Inflammatory Profiles in OSCC

The inflammatory profiles are much more heterogeneous in established OSCC than in precursor lesions. The studies that have characterized the immune landscape of SCC at various sites are in overall agreement that OSCC and other HPV-negative SCC are most likely to exhibit unfavorable non-Th1 profiles, as illustrated in several recent publications mentioned here. Investigation of head and neck and other SCC using the Cancer Genome Atlas (TCGA) identified at least 6 immune profiles depending upon the activity in angiogenesis, inflammation, reactive stroma, T cell, IFN-gamma, TGF-beta, and differentiation pathways ranging from favorable Th1-dominated to immunosuppressive type, immune cold type, and additional intermediate types [144]. As predicted, tumors associated with high CD8^+^, NK cell, and IFN-gamma presence, i.e. Th1 profile, had the best prognosis [144]. Some studies have found a Th17 profile in subsets of head and neck and OSCC TME and in the peripheral blood of such patients, and this profile was associated with worse prognosis than Th1 type. For comparison, HPV-related SCC of either head and neck or uterine cervix origin were much more likely than HPV-negative SCC to present with the Th1-dominant profile (presumably, due to the presence of viral antigens), although unfavorable profiles, especially the immunosuppressive type, overlapping with those found in HPV-negative SCC were also identified [145]. Single-cell transcriptional analysis also revealed significant differences between HPV-related and HPV-negative HNSCC immune profiles, especially in the B cell, myeloid cell, and conventional CD4^+^ T cell populations [145], perhaps in part because the vast majority of HPV-related head and neck carcinomas, unlike HPV-negative OSCC, arise in the MALT of the oropharynx. Yet, much remains unknown about exactly how these profiles become established, or how consistent they are throughout the mass. Given the plasticity of myeloid and T cell populations [145], variation in tumor location, the ongoing recruitment of new leukocytes, conditions in different areas of the mass, as well as evolution of microbial colonization, immune profiles may vary throughout the tumor.

Notably, OSCC and other mucosal cancers sometimes contain newly-developed tertiary lymphoid structures (TLS). TLS consist of organized B cell follicles, T cell-rich areas, and antigen presenting cells, with or without high endothelial venules [146–150], and are distinct from the secondary lymphoid structures called MALT. TLS presence in various cancers generally correlates with improved patient survival, although in OSCC, there is more heterogeneity than in other cancers for unknown reasons [148]. Moreover, TLS in the oral mucosa and other sites are not unique to cancers, but are also found in infections and non-infectious chronic inflammatory disorders [147, 151].

Recent advances in immunotherapies aimed at improving T cell responses are making progress in head and neck cancer management, especially therapies focused on tumor cell-T cell interactions [152–156]. The success of Th1-based antitumor response depends in part on the antigens expressed by the tumor cells. The co-localization of microorganisms and the malignant cells in OSCC with a never healing surface presents a range of potential targets for the immune response, and contributes to the chronically inflamed, smoldering wound-like environment of OSCC and of other digestive tract carcinomas unable to reestablish normal barrier. While TLR ligands are the most obvious initiators of inflammatory mechanisms in macrophages and dendritic cells that are followed by the adaptive response, the antigen specificity of the adaptive immune responses within the microenvironment of OSCC and other surface carcinomas is poorly characterized. There is increasing evidence from various carcinoma studies of peripheral blood cells and circulating antibodies that T and B cells respond to shared (i.e. expressed by both normal and tumor cells) and to tumor-specific antigens (i.e. resulting from mutations), which can predict better prognosis in some cancers [157, 158]. However, intratumoral carcinoma cell-specific responses are often low. For example, only 0–10% of intratumoral CD8^+^ T cells in ovarian and colon carcinoma patients could recognize tumor antigens [159]. Moreover, tumor vaccine trials using a widely expressed shared antigen (MUC1) in patients with colorectal adenoma showed that over 50% of patients did not respond to the vaccine, which correlated with high numbers of circulating MDSC [160], revealing that interference with anti-tumor immune responses may develop already at the pre-cancerous stage. Notably, carcinoma cell death can also benefit the tumor as a whole because of the release of DAMP and ATP (discussed more below) with pro-tumor effects on the microenvironment and the surviving tumor cells.

NK-based therapies are also under investigation, as NK cells can kill tumor cells that lack MHC class I [161]. TLR ligands are also being tested for the ability to enhance T cell-mediated antitumor response because of their stimulatory effects on DC maturation, migration, and antigen presentation. Yet, interference with cytotoxic T cell, NK cell, and IFN-gamma antitumor responses, many of which are connected to TLR activities, remains a significant hurdle to overcome. Particularly relevant to this review is that conditions in OSCC and other surface-associated carcinomas generate—and selectively amplify—cell subsets and molecules utilized in pro-homeostatic processes, such as non-Th1 innate and adaptive immune cells, myeloid suppressor cells, regulatory T cells, and products of immune and non-immune cells in the TME that interfere with antitumor immune responses.

The apparent switch from more destructive immune profiles in premalignant lesions to the predominantly immunosuppressive immune profiles in OSCC point to the possibility that TLR may be essential to the process, given their functions in non-cancerous conditions.

#### Cell Surface TLR and OSCC TME: Interference With Successful Antitumor Responses

Evidence indicates that cell-surface TLR are important contributors to the success of carcinogenesis. In addition to immune system and other stromal cells, functional TLR are also expressed by malignant epithelial cells, including OSCC, and participate in the biology of carcinomas. So far, the best characterized PRR in carcinoma cells are TLR2 and TLR4. The outcomes of carcinoma cell TLR signaling affect inflammatory conditions as well as have tumor cell-intrinsic effects unrelated to inflammation, because TLR activate NF-kB [96, 110, 162] and ERK1/2 MAPK [96] pathways in OSCC cells. Examples of cytokines and growth factors induced by TLR2 and/or TLR4 in OSCC cells include IL-1, IL-6, GM-CSF, TNF-alpha, CCL2, CCL20, CXCL8, and VEGF [96, 109, 110], which contribute to the inflammatory environment, vascularization, and tumor cell properties. TLR5 expression has been identified in OSCC, although little is known about its role. There is evidence from animal studies that TLR5 signaling at mucosal surfaces promotes systemic inflammation dependent upon tumor cell- and leukocyte-derived IL-6, and involves MDSC and gamma-delta T cells, driving progression of extra-intestinal cancers. These observations are supported by human data [163].

The roles of immune and carcinoma cell surface TLR2 and TLR4 in inflammation and immunosuppression are discussed first, followed by non-inflammatory cell-intrinsic carcinoma cell TLR functions.

##### Monocytes-Macrophages-M-MDSC

**Figure 3:**
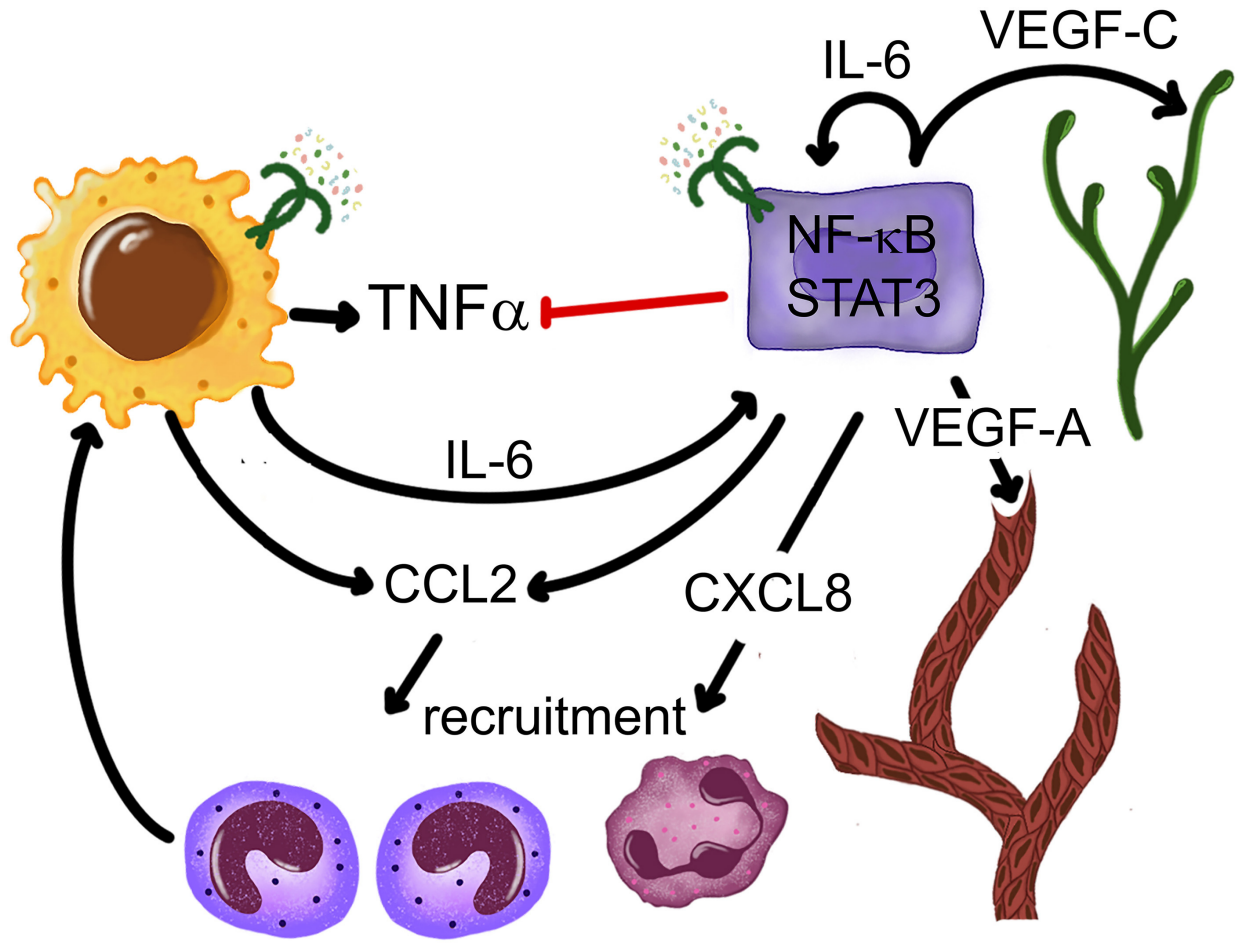
Outcomes of interactions between OSCC cells, monocytes and a TLR4 ligand *E. coli* LPS. OSCC cells selectively inhibit LPS-induced TNF-alpha production in monocytes-macrophages without disrupting IL-6. OSCC and monocyte-macrophage-derived IL-6 activates STAT3 in most cells. LPS-induced CCL2 and CXCL8 recruit monocytes and neutrophils IL-6-induced STAT3 activation upregulates VEGF-A and VEGF-C production, stimulating vasculogenesis and lymphangiogenesis, respectively. References: Kurago et al. [109], Lam-ubol et al. [179], Palani et al. [96], Rajarathnam et al. [180], and Shinriki et al. [181].

This is a particularly well-studied group of cells in the TME so far. A variety of monocyte and macrophage phenotypes are highly represented in all of the described types of carcinoma immune profiles. A subset of macrophages derives from a proliferating pool of original resident macrophages, while a continually increasing pool comes from newly recruited monocytes [164]. Monocytes are actively recruited to tumors, including OSCC, by several chemokines [165, 166], where they proliferate and differentiate with remarkable plasticity. Particularly relevant to this recruitment are the NF-kB-dependent CCL2-5, inducible in the TME and other inflammatory conditions via TLR [167–169]. TLR2/1 and TLR4 activities alone or combined also stimulate CCL2 production in OSCC cells [96, 109]. TLR stimuli contribute to the spectrum of TME monocyte-lineage cells from M1 to M2 with intermediate phenotypes [170] and to M-MDSC [171], but the pro-tumor M2 (also referred to as tumor associated macrophages, TAM) and M-MDSC are dominant in established OSCC and in the circulation of patients with cancer [110, 161, 172]. LPS triggers metabolic reprogramming in macrophages [173, 174], including TAM, which conditions the TME to support tumor growth [170]. OSCC cells also help to support TME macrophages, because TLR4 stimulate OSCC cells to secrete GM-CSF [110], which is an important factor for the development and maintenance of macrophages [175]. As discussed earlier, TLR contribute to the evolution from M1 to M2 in other settings, which are normally important to recover homeostasis. However, as malignant epithelial cells in OSCC cannot repair the barrier, the M2 and M-MDSC continue to receive TLR- and cytokine-mediated stimulation, produce IL-6, IL-10, TGF-beta, and other factors that suppress antitumor responses, and also support cancer cells in other ways, such as secrete epidermal growth factor (EGF) and VEGFA [170, 176–178]. M-MDSC that receive TLR2 stimuli in combination with Th1 cytokine IFN-gamma become inducible nitric oxide synthase (iNOS)^+^ macrophages that impede proliferation of CD8^+^ T cells [172], thus compromising anti-tumor cytotoxicity. Selected aspects of LPS-induced effects on OSCC and monocytes/macrophages are illustrated in Figure 3.

As mentioned previously, TLR ligand-stimulated monocytes and macrophages are a major source of TNF-alpha and IL-6, key factors in TME that are also inducible in other cells including OSCC [96, 179]. Similar to other factors generated during inflammation (ROS, for example), TNF-alpha has tumor-destructive and tumor-promoting properties, which appear to depend on the receptor (TNFR1 vs. TNFR2) [182] and the amount of TNF [123, 127, 183, 184]. Low TNF concentrations produce pro-tumor activities in the TME via NF-kB-mediated induction of cytoprotective and angiogenic factors IL-6 and VEGF, differentiation of mesenchymal stem cells into endothelial cells, increase in vascular permeability, recruitment of neutrophils and monocytes, monocyte differentiation into MDSC, and promotion of Tregs [184]. Anti-tumor activities involve relatively high TNF concentrations and binding to TNFR1, which mediates stromal and tumor cell apoptosis, microvasculature collapse, and increased T and NK cell cytotoxicity [183, 184], as well as prevents the transition from M1 to M2 [111].

The TME is equipped to control TNF-alpha production. Negative regulation of TNF-alpha levels involves TLR and adenosine derived from ATP. ATP levels in the TME of solid cancers are elevated due to cell death, inflammation and hypoxia [185, 186], and may also come from the colonizing microorganisms. Although not tested using OSCC-associated microbiome, a variety of human bacterial pathogens are known to produce and release ATP during growth [187]. ATP is dephosphorylated by cell-surface enzymes CD39 and CD73 on immune system, tumor, and other cells, which then results in often micro-molar adenosine concentrations in the TME [185, 188]. Cell surface TLR activity induced by MAMP or DAMP (such as versican and HMGB1) leads to increased expression of inhibitory A2a and A2b adenosine receptors through which adenosine selectively inhibits TNF-alpha production in monocytes/macrophages. Our own studies showed that in the presence of OSCC cells, monocytes altered their response to *E. coli* LPS by markedly reducing TNF-alpha production, but maintained high levels of intracellular and secreted cytoprotective IL-6 (Figure 3), and in the process also acquired intermediate or non-classical monocyte phenotype [179] representative of anti-inflammatory monocytes [189]. The most likely candidate that explains suppression of TNF-alpha is adenosine, as these OSCC cells express CD39 and CD73. On the other hand, OSCC cells may also be subject to effects of adenosine, because the inhibitory A2a AR are upregulated by TLR2 activity in OSCC cells and are able to signal via the MAPK ERK1/2 pathway [96], while A2b AR were shown to promote OSCC cell proliferation *in vitro* [190].

The critical protumor role of IL-6 in cancer, including OSCC, is well-known [114, 191–193]. Besides monocytes and macrophages, other TME-associated cell types, including OSCC cells, produce it (and other IL-6-family cytokines) in response to TLR ligands and other NF-kB-inducing inflammatory mediators. As mentioned previously, by activating STAT3 in immune system cells IL-6 contributes to immunosuppression. This effect is amplified by IL-10, which is also abundant in OSCC TME [142]. A recent study clearly linked TLR-triggered NF-kB activation with induction of STAT3-mediated immunosuppression in TME. Tang et al. showed that cancer cell-derived DAMP versican activated TLR2 in TME cells, and the induced IL-6 and IL-10 synergistically caused STAT3 activation in intratumoral DC, which resulted in their dysfunction in terms of directing Th1 and cytotoxic antitumor response [194]. This is yet another example of TLR2 role in tumor-related immunosuppression.

Besides suppressing toxic immune responses, STAT3 functions as an oncogene in malignant cells and is a key factor that links inflammation and cancer [114]. Activated STAT3 helps tumor cells to proliferate, survive insults, resist noxious chemicals and potent inflammatory mediators, and it supports aerobic glycolysis, reduces reactive oxygen species, and protects tumor-initiating cells also known as cancer stem-like cells (CSC) [195]. Our studies showed that OSCC cells did not have constitutively activated STAT3, but soluble factors generated in response to TLR4 stimuli either in monocytes, in monocyte-OSCC co-cultures, or in some OSCC cell lines, were responsible for OSCC STAT3 activation; blocking studies indicated that IL-6 was one of the responsible factors [109]. In addition to cytokines, growth factors acting on their receptors, such as epidermal growth factor receptor (EGFR), also activate STAT3 [196], and macrophages are an important source of EGF in OSCC [197]. TLR and STAT3 activity in carcinoma cells [109] promotes TME vasculogenesis and lymphangiogenesis due to production of VEGF-A and VEGF-C, respectively [181, 198, 199].

##### Neutrophils (Polymorphonuclear Leukocytes, PMN)

Besides monocyte-derived M-MDSC, PMN-derived subsets are also important contributors to the tumor microenvironment and are recruited in response to TLR signals. TLR-induced chemokines including NF-kB-dependent CXCLs 1, 2, 8 (and other factors) recruit neutrophils via CXCR1 and CXCR2 [180]. OSCC cells contribute to neutrophil recruitment because they produce CXCL8 when stimulated by TLR2/1 or TLR4 ligands [96, 110]. A recent study using CIBERSORT (Cell type Identification By Estimating Relative Subsets Of known RNA Transcripts) method showed that among leukocyte gene signatures found in various cancers, including HNSCC, high neutrophil presence carried the most significant adverse prognosis [200], and neutrophil contributions were also noted in oral cancer [201–203]. Neutrophils are recruited to sites of mucosal surface breach via chemokine receptor signals. Like other immune system cells they express functional cell-surface and intracellular TLR [204, 205], and TLR4 activity contributes to neutrophil survival [206]. Similar to monocytes-macrophages, neutrophils can promote carcinogenesis as type 2 (N2) or as related populations called PMN-MDSC or GR-MDSC by impeding antitumor immune responses [207]. The role of neutrophil TLR in protumor functions of these cell subsets is not clear. However, in inflammatory bowel disease (IBD), which is a precursor to IBD-associated colon cancer, PMN were found to accumulate in large numbers and to release myeloperoxidase (MPO) along with other enzymes [208]. The study team demonstrated that MPO-catalyzed reactive oxygen species (ROS) caused intestinal epithelial injury and contributed to the impaired wound healing in this model. The soluble factors released by activated neutrophils, and potentially induced in a TLR-dependent manor, are among those associated with carcinogenesis, including IL-1, IL-6, IL-10, TGF-beta, factors important for angiogenesis, and ROS [205, 207]. However, whether TLR signals specifically contribute to the transition of neutrophils to one of the pro-tumor phenotypes has not been determined. It is also unclear how infiltration of tumors by neutrophil subsets affects the associated microbial communities.

##### Other TME Cells

TLR signals also affect T and B cell phenotypes and function in the TME. The immunosuppression generated by monocyte, macrophage and neutrophil subsets is enhanced because the effector Treg cell subset is highly enriched in OSCC and other carcinomas that express the NF-kB-dependent chemokine CCL20, a factor that is also important for homeostasis [209–211]. CCL20 is significantly increased in the saliva and tissues of OSCC patients [212], and is often secreted by OSCC cells, especially in the presence of TLR ligands [109]. Moreover, TLR2 and A2a AR together contribute to the induction of Treg [213]. That said, data on the prognostic impact of Treg in TME of OSCC appear to be controversial, as both pro- and anti-tumor Treg activities are proposed in different studies [214, 215]. In addition, CCL20 also attracts other immune system cells that express its receptor CCR6, such as Th17 [216], which contribute to a pro-tumor TME in various cancers. However, the mechanisms of Th17 activity in OSCC are unknown. Even B cell TLR activity was shown to trigger their regulatory function and suppress both Th1 and TH17 cells [116], although much remains unknown regarding Breg functions in OSCC.

Other tumor stromal cells, such as fibroblasts and endothelial cells also respond to TLR ligands, most consistently those specific for TLR2 and TLR4, which contributes to inflammation, tissue remodeling, and angiogenesis [217], though studies of these functions in TME are limited. There is evidence that stromal and cancer-associated fibroblasts promote tumorigenesis via TLR4/MyD88 signaling [218]. Little is known about endothelial cell TLR function in the TME.

In summary, accumulating data reveal the multifaceted contributions of cell surface TLR, especially TLR2, to a cancer cell-friendly microenvironment by acting on stromal and carcinoma cells with a profound impact on the quality of inflammation.

#### Non-inflammatory Cell-Intrinsic Effects of TLR in OSCC Cells

**Figure 4:**
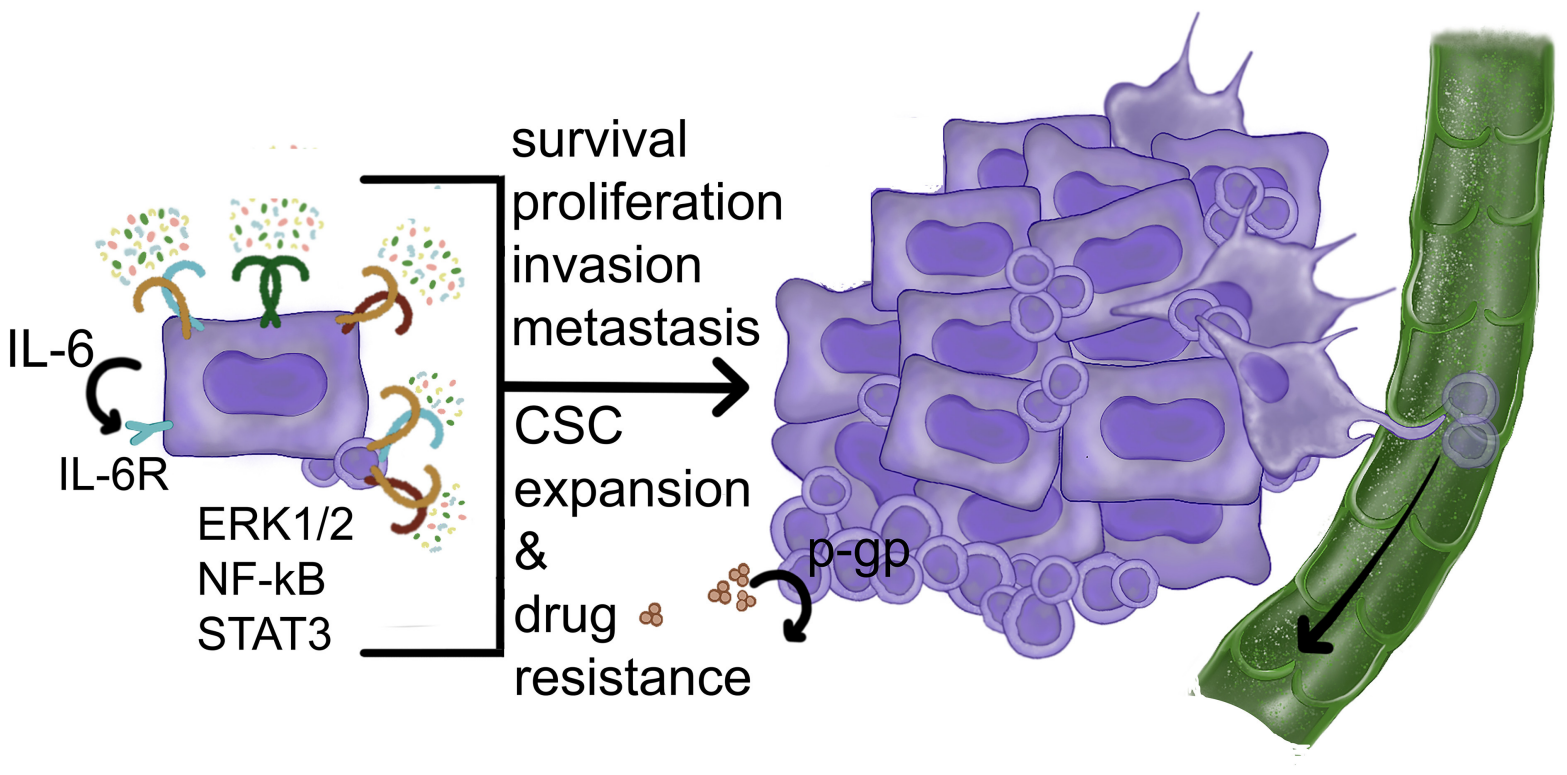
Carcinoma cell-intrinsic pro-tumor effects of TLR activities. TLR ligands activate NF-kB and ERK1/2 MAPK pathways leading to increased proliferation, improved survival, and resistance to drugs. The induction of STAT3-activating factors (such as IL-6) can also contribute to CSC expansion and survival. So far the role of p-gp has been demonstrated in normal stem cells. References: Frank et al. [219], Kurago et al. [109], Palani et al. [96], Shinriki et al. [181], Szczepanski et al. [110], and Yeh et al. [162].

Multiple carcinoma cell-intrinsic effects due to TLR activities are possible via TLR-mediated signals through NF-kB and MAPK pathways along with induction of STAT3-activating factors with benefits for tumor growth and survival. TLR2 function in carcinoma cells has been studied the most so far (discussed below) (Figure 4). Data on flagellin-induced TLR5 signaling in OSCC and HPV-negative oropharyngeal SCC are limited and somewhat mixed, as despite high TLR5 expression revealed by immunohistochemistry, NF-kB activation was not detected in two HPV-negative SCC cell lines [102, 220, 221]. Immunohistochemistry-based studies in tongue SCC show a range of TLR2, 4 and 5 expression in OSCC cells and suggest that the amount of TLR expression in carcinoma cells may positively correlate with more advanced tumors and worse outcomes [103, 222, 223]. However, other studies are contradictory [224]. Other approaches are necessary to resolve the controversy.

The most detailed account of carcinoma cell-intrinsic TLR2 function was described in gastric adenocarcinoma cells that expressed high levels of TLR2, resulting in increased ligand-induced cell proliferation and survival independent of inflammation. These functions were related to several upregulated anti-apoptotic genes and cell cycle progression/proliferation genes, as well as decreased expression of tumor suppressor genes, first identified in mouse models and verified in human samples that expressed high levels of TLR2 [225]. These results have important implications for treatment strategies, because more than 50% of intestinal type gastric adenocarcinomas irrespective of ethnicities or geographic distribution, are TLR2-high [225]. Moreover, TLR2 activation in human gastric cancer cells also affected cell metabolism, leading to induction of both OXPHOS and glycolysis with a bias toward glycolysis [226]. In addition, there was a positive correlation between superoxide dismutase (SOD) and TLR2 expression with associated poor patient survival [226]. It is possible that gastric adenocarcinoma responses to TLR activation are distinct from those in other carcinomas because of the causative role the bacterium *Helicobacter pylori* plays in gastric carcinogenesis. However, cell-intrinsic benefits of TLR2 activity have also been described in carcinomas of the intestine, breast and liver [89], ovary [227, 228], and OSCC.

In OSCC cells, most reports show TLR2- or TLR4-mediated increase in proliferation and survival [96, 107, 108, 110]. Our own studies connected TLR2-induced OSCC cell proliferation and resistance to apoptosis to the ERK1/2 MAPK pathway [96]. Notably, the TLR2/1 and TLR2/6 heterodimers were both active in OSCC cells that expressed more TLR2 than TLR4 based upon quantitative PCR analysis. Our study also revealed that only one out of five evaluated OSCC cell lines expressed more TLR4 than TLR2 and showed very little response to TLR2 stimuli [96]. In addition, TLR/MyD88 activation in OSCC cells by periodontal pathogens was shown to trigger integrin/FAK signaling, which then increased the aggressive behavior of OSCC cells [28]. Considering that TLR activity induces metabolic changes with increase in glycolysis in macrophages and gastric carcinoma cells, it is theoretically possible that this mechanism may operate in OSCC cells.

As mentioned earlier, activated TLR2 or TLR4 in OSCC can trigger the production of IL-6 and other STAT3-activating factors that then act in autocrine fashion on the OSCC receptors, which is another major cell-intrinsic pro-tumor benefit of TLR activity. Whether activated STAT3 in OSCC cells can stimulate TLR2 expression in the way it does in gastric carcinoma cells [229] is not known. How TLR expression is regulated in OSCC cells is another important question to investigate.

STAT3 is essential for the maintenance of a stem cell phenotype [230], and accumulating evidence suggests that TLR signaling is also important in undifferentiated epithelial cells and cancer stem-like cells (CSC) [89] because of the downstream NF-kB activity. NF-kB has been implicated in regulating stem-like properties and their self-renewal in various carcinomas, including HNSCC. HNSCC cultured in conditions that enrich stemness showed activated NF-kB with reduced levels of negative regulators of TLR signaling [162]. In addition, NF-kB targets COX2/PGE2 were reported to maintain stem-like properties in gastric adenocarcinoma [231]. Another pathway shown to regulate HNSCC CSC reprogramming involves c-FOS [232], which we found to be activated by TLR2 ligands via MAPK ERK1/2 in OSCC cells. We also noticed that immunohistochemical staining of OSCC specimens showed more intense labeling of smaller undifferentiated carcinoma cells than the large well-differentiated cells [96]. CSC are known to be more resistant than other subsets to chemotherapy, so that TLR stimuli may be contributing to this resistance. For example, TLR4 activation was shown to protect OSCC cells from cisplatin toxicity [110]. Although not tested in carcinoma cells, evidence also points to a role for TLR2 in protecting cells from genotoxic insults by limiting damaging inflammation and maintaining the assembly of p-glycoprotein (p-gp), a drug efflux transporter pump, which is expressed in myeloid and epithelial cells among other cell types [219]. The mechanisms of cancer cell resistance to therapy may involve NF-kB-mediated and MAPK-mediated activities generated by TLR and NF-kB-activating inflammatory factors, along with subsequent activation of STAT3-mediated maintenance of CSC. Figure 4 summarizes several TLR-mediated cell-intrinsic effects on carcinoma cells.

## Conclusions

The comprehensive approach to this review was used in order to develop a more complete landscape of oral carcinogenesis in the context of microbial colonization and cell-surface PRR designed to perform many functions to protect the host from infections and dangerous runaway immune reactions. The evidence discussed here indicates that in the slow process of carcinogenesis, the initially destructive properties of cell-surface TLR activation are tightly controlled and usually short-lived, and anti-destructive mechanisms take over, in a way consistent with the process in infections or injury. The overwhelming input of many DAMP- and MAMP-mediated signals into malignant cells, stromal immune cells and non-immune cells, works against the few available toxic mechanisms that are necessary to destroy the malignant cells, and complicates the search for effective treatments. A more comprehensive approach to patient treatment throughout the process of carcinogenesis that incorporates better understanding of TLR contributions to the evolving or established TME has the potential to improve patient outcomes.

## Author Contributions

ZK: conceptual design, acquisition and analysis of relevant publications, outline for potential figures, and writing of the manuscript. JL: acquisition and analysis of relevant literature, design and production of figures, and writing portions of the manuscript. All authors contributed to the article and approved the submitted version.

## Conflict of Interest

The authors declare that the research was conducted in the absence of any commercial or financial relationships that could be construed as a potential conflict of interest.

## Publisher's Note

All claims expressed in this article are solely those of the authors and do not necessarily represent those of their affiliated organizations, or those of the publisher, the editors and the reviewers. Any product that may be evaluated in this article, or claim that may be made by its manufacturer, is not guaranteed or endorsed by the publisher.

## References

[1] Neville B Damm DD Allen C Chi A. Oral and Maxillofacial Pathology. 4th ed. St. Louis, MO: Elsevier (2016). p. 355–89.

[2] El-Naggar AK Chan JKC Grandis JR Takata T Slootweg PJ. WHO Classification of Head and Neck Tumors. 4th ed. Lyon: International Agency for Research on Cancer (2017), pp. 109–115.

[3] Janz TA Graboyes EM Nguyen SA Ellis MA Neskey DM Harruff EE. A Comparison of the NCDB and SEER database for research involving head and neck cancer. Otolaryngol Head Neck Surg. (2019) 160:284–94. 10.1177/0194599818792205 30129822

[4] Nanci A. Ten Cate's Histology 9th ed. St. Louis, MO: Elsevier (2018).

[5] Effiom OA Adeyemo WL Omitola OG Ajayi OF Emmanuel MM Gbotolorun OM. Oral squamous cell carcinoma: a clinicopathologic review of 233 cases in Lagos, Nigeria. J Oral Maxillofac Surg. (2008) 66:1595–9. 10.1016/j.joms.2007.12.025 18634945

[6] Gupta N Gupta R Acharya AK Patthi B Goud V Reddy S. Changing trends in oral cancer - a global scenario. Nepal J Epidemiol. (2016) 6:613–9. 10.3126/nje.v6i4.17255 28804673 PMC5506386

[7] Jacobs CD Barbour AB Mowery YM. The relative distribution of oral cancer in the United States by subsite. Oral Oncol. (2019) 89:56–8. 10.1016/j.oraloncology.2018.12.017 30732959

[8] Brandner JM Zorn-Kruppa M Yoshida T Moll I Beck LA De Benedetto A. Epidermal tight junctions in health and disease. Tissue Barriers. (2015) 3:e974451. 10.4161/21688370.2014.974451 25838981 PMC4372028

[9] Crawford M Dagnino L. Scaffolding proteins in the development and maintenance of the epidermal permeability barrier. Tissue Barriers. (2017) 5:e1341969. 10.1080/21688370.2017.1341969 28665776 PMC5788441

[10] Gopinath D Menon RK Wie CC Banerjee M Panda S Mandal D. Differences in the bacteriome of swab, saliva, and tissue biopsies in oral cancer. Sci Rep. (2021) 11:1181. 10.1038/s41598-020-80859-0 33441939 PMC7806708

[11] Human Microbiome Project C. Structure, function and diversity of the healthy human microbiome. Nature. (2012) 486:207–14. 10.1038/nature11234 22699609 PMC3564958

[12] Sender R Fuchs S Milo R. Are we really vastly outnumbered? Revisiting the ratio of bacterial to host cells in humans. Cell. (2016) 164:337–40. 10.1016/j.cell.2016.01.013 26824647

[13] Baker JL Bor B Agnello M Shi W He X. Ecology of the oral microbiome: beyond bacteria. Trends Microbiol. (2017) 25:362–74. 10.1016/j.tim.2016.12.012 28089325 PMC5687246

[14] Mougeot JC Stevens CB Morton DS Brennan MT Mougeot FB. Oral microbiome and cancer therapy-induced oral mucositis. J Natl Cancer Inst Monogr. (2019) 2019:lgz002. 10.1093/jncimonographs/lgz002 31425594

[15] Sami A Elimairi I Stanton C Ross RP Ryan CA. The role of the microbiome in oral squamous cell carcinoma with insight into the microbiome-treatment axis. Int J Mol Sci. (2020) 21:8061. 10.3390/ijms21218061 33137960 PMC7662318

[16] Sultan AS Kong EF Rizk AM Jabra-Rizk MA. The oral microbiome: a lesson in coexistence. PLoS Pathog. (2018) 14:e1006719. 10.1371/journal.ppat.1006719 29370304 PMC5784999

[17] Dzutsev A Goldszmid RS Viaud S Zitvogel L Trinchieri G. The role of the microbiota in inflammation, carcinogenesis, and cancer therapy. Eur J Immunol. (2015) 45:17–31. 10.1002/eji.201444972 25328099

[18] Wu J Peters BA Dominianni C Zhang Y Pei Z Yang L. Cigarette smoking and the oral microbiome in a large study of American adults. ISME J. (2016) 10:2435–46. 10.1038/ismej.2016.37 27015003 PMC5030690

[19] Fan X Peters BA Jacobs EJ Gapstur SM Purdue MP Freedman ND. Drinking alcohol is associated with variation in the human oral microbiome in a large study of American adults. Microbiome. (2018) 6:59. 10.1186/s40168-018-0448-x 29685174 PMC5914044

[20] Sakamoto H Naito H Ohta Y Tanakna R Maeda N Sasaki J. Isolation of bacteria from cervical lymph nodes in patients with oral cancer. Arch Oral Biol. (1999) 44:789–93. 10.1016/S0003-9969(99)00079-5 10530911

[21] Amer A Galvin S Healy CM Moran GP. The microbiome of potentially malignant oral leukoplakia exhibits enrichment for Fusob*acterium, Leptotrichia, Campylobacter*, and *Rothia* species. Front Microbiol. (2017) 8:2391. 10.3389/fmicb.2017.02391 29250055 PMC5717034

[22] Huybrechts I Zouiouich S Loobuyck A Vandenbulcke Z Vogtmann E Pisanu S. The human microbiome in relation to cancer risk: a systematic review of epidemiologic studies. Cancer Epidemiol Biomarkers Prev. (2020) 29:1856–68. 10.1158/1055-9965.EPI-20-0288 32727720 PMC7541789

[23] Rubinstein MR Baik JE Lagana SM Han RP Raab WJ Sahoo D. Fusobacterium nucleatum promotes colorectal cancer by inducing Wnt/beta-catenin modulator annexin A1. EMBO Rep. (2019) 20:e47638. 10.15252/embr.201847638 30833345 PMC6446206

[24] Rubinstein MR Wang X Liu W Hao Y Cai G Han YW. Fusobacterium nucleatum promotes colorectal carcinogenesis by modulating E-cadherin/beta-catenin signaling via its FadA adhesin. Cell Host Microbe. (2013) 14:195–206. 10.1016/j.chom.2013.07.012 23954158 PMC3770529

[25] Fitzsimonds ZR Rodriguez-Hernandez CJ Bagaitkar J Lamont RJ. From beyond the pale to the pale riders: the emerging association of bacteria with oral cancer. J Dent Res. (2020) 99:604–12. 10.1177/0022034520907341 32091956 PMC7243420

[26] Brennan CA Garrett WS. Fusobacterium nucleatum - symbiont, opportunist and oncobacterium. Nat Rev Microbiol. (2019) 17:156–66. 10.1038/s41579-018-0129-6 30546113 PMC6589823

[27] Binder Gallimidi A Fischman S Revach B Bulvik R Maliutina A Rubinstein AM. Periodontal pathogens *Porphyromonas gingivalis* and *Fusobacterium nucleatum* promote tumor progression in an oral-specific chemical carcinogenesis model. Oncotarget. (2015) 6:22613–23. 10.18632/oncotarget.4209 26158901 PMC4673186

[28] Kamarajan P Ateia I Shin JM Fenno JC Le C Zhan L. Periodontal pathogens promote cancer aggressivity via TLR/MyD88 triggered activation of Integrin/FAK signaling that is therapeutically reversible by a probiotic bacteriocin. PLoS Pathog. (2020) 16:e1008881. 10.1371/journal.ppat.1008881 33002094 PMC7529280

[29] Zackular JP Baxter NT Iverson KD Sadler WD Petrosino JF Chen GY. The gut microbiome modulates colon tumorigenesis. MBio. (2013) 4:e00692-13. 10.1128/mBio.00692-13 24194538 PMC3892781

[30] Stashenko P Yost S Choi Y Danciu T Chen T Yoganathan S. The oral mouse microbiome promotes tumorigenesis in oral squamous cell carcinoma. mSystems. (2019) 4:e00323-19. 10.1128/mSystems.00323-19 31387932 PMC6687944

[31] Khosravi A Yanez A Price JG Chow A Merad M Goodridge HS. Gut microbiota promote hematopoiesis to control bacterial infection. Cell Host Microbe. (2014) 15:374–81. 10.1016/j.chom.2014.02.006 24629343 PMC4144825

[32] Healy CM Moran GP. The microbiome and oral cancer: more questions than answers. Oral Oncol. (2019) 89:30–3. 10.1016/j.oraloncology.2018.12.003 30732955

[33] Yost S Stashenko P Choi Y Kukuruzinska M Genco CA Salama A. Increased virulence of the oral microbiome in oral squamous cell carcinoma revealed by metatranscriptome analyses. Int J Oral Sci. (2018) 10:32. 10.1038/s41368-018-0037-7 30420594 PMC6232154

[34] Lamont RJ Koo H Hajishengallis G. The oral microbiota: dynamic communities and host interactions. Nat Rev Microbiol. (2018) 16:745–59. 10.1038/s41579-018-0089-x 30301974 PMC6278837

[35] Alnuaimi AD Ramdzan AN Wiesenfeld D O'Brien-Simpson NM Kolev SD Reynolds EC. Candida virulence and ethanol-derived acetaldehyde production in oral cancer and non-cancer subjects. Oral Dis. (2016) 22:805–14. 10.1111/odi.12565 27495361

[36] Alnuaimi AD Wiesenfeld D O'Brien-Simpson NM Reynolds EC McCullough MJ. Oral Candida colonization in oral cancer patients and its relationship with traditional risk factors of oral cancer: a matched case-control study. Oral Oncol. (2015) 51:139–45. 10.1016/j.oraloncology.2014.11.008 25498921

[37] Pulendran B Artis D. New paradigms in type 2 immunity. Science. (2012) 337:431–5. 10.1126/science.1221064 22837519 PMC4078898

[38] Fitzgerald KA Kagan JC. Toll-like receptors and the control of immunity. Cell. (2020) 180:1044–66. 10.1016/j.cell.2020.02.041 32164908 PMC9358771

[39] Oosting M Cheng SC Bolscher JM Vestering-Stenger R Plantinga TS Verschueren IC. Human TLR10 is an anti-inflammatory pattern-recognition receptor. Proc Natl Acad Sci USA. (2014) 111:E4478–84. 10.1073/pnas.1410293111 25288745 PMC4210319

[40] Nakayama H Kurokawa K Lee BL. Lipoproteins in bacteria: structures and biosynthetic pathways. FEBS J. (2012) 279:4247–68. 10.1111/febs.12041 23094979

[41] Kovacs-Simon A Titball RW Michell SL. Lipoproteins of bacterial pathogens. Infect Immun. (2011) 79:548–61. 10.1128/IAI.00682-10 20974828 PMC3028857

[42] Kurokawa K Kim MS Ichikawa R Ryu KH Dohmae N Nakayama H. Environment-mediated accumulation of diacyl lipoproteins over their triacyl counterparts in *Staphylococcus aureus*. J Bacteriol. (2012) 194:3299–306. 10.1128/JB.00314-12 22467779 PMC3434734

[43] Wolf AJ Underhill DM. Peptidoglycan recognition by the innate immune system. Nat Rev Immunol. (2018) 18:243–54. 10.1038/nri.2017.136 29292393

[44] Kondo T Kawai T Akira S. Dissecting negative regulation of toll-like receptor signaling. Trends Immunol. (2012) 33:449–58. 10.1016/j.it.2012.05.002 22721918

[45] Kim S Karin M. Role of TLR2-dependent inflammation in metastatic progression. Ann N Y Acad Sci. (2011) 1217:191–206. 10.1111/j.1749-6632.2010.05882.x 21276007 PMC4383094

[46] Yang S Liu L Xu D Li X. The relationship of the TLR9 and TLR2 genetic polymorphisms with cervical cancer risk: a meta-analysis of case-control studies. Pathol Oncol Res. (2020) 26:307–15. 10.1007/s12253-018-0465-x 30215163

[47] Kim S Takahashi H Lin WW Descargues P Grivennikov S Kim Y. Carcinoma-produced factors activate myeloid cells through TLR2 to stimulate metastasis. Nature. (2009) 457:102–6. 10.1038/nature07623 19122641 PMC2746432

[48] Vijayan A Rumbo M Carnoy C Sirard JC. Compartmentalized antimicrobial defenses in response to flagellin. Trends Microbiol. (2018) 26:423–35. 10.1016/j.tim.2017.10.008 29173868

[49] Karin M Lawrence T Nizet V. Innate immunity gone awry: linking microbial infections to chronic inflammation and cancer. Cell. (2006) 124:823–35. 10.1016/j.cell.2006.02.016 16497591

[50] Foster SL Hargreaves DC Medzhitov R. Gene-specific control of inflammation by TLR-induced chromatin modifications. Nature. (2007) 447:972–8. 10.1038/nature05836 17538624

[51] Hamerman JA Pottle J Ni M He Y Zhang ZY Buckner JH. Negative regulation of TLR signaling in myeloid cells–implications for autoimmune diseases. Immunol Rev. (2016) 269:212–27. 10.1111/imr.12381 26683155 PMC4703580

[52] Rakoff-Nahoum S Paglino J Eslami-Varzaneh F Edberg S Medzhitov R. Recognition of commensal microflora by toll-like receptors is required for intestinal homeostasis. Cell. (2004) 118:229–41. 10.1016/j.cell.2004.07.002 15260992

[53] Lin ZQ Kondo T Ishida Y Takayasu T Mukaida N. Essential involvement of IL-6 in the skin wound-healing process as evidenced by delayed wound healing in IL-6-deficient mice. J Leukoc Biol. (2003) 73:713–21. 10.1189/jlb.0802397 12773503

[54] Belkaid Y Artis D. Immunity at the barriers. Eur J Immunol. (2013) 43:3096–7. 10.1002/eji.201344133 24166766 PMC4820323

[55] Belkaid Y Naik S. Compartmentalized and systemic control of tissue immunity by commensals. Nat Immunol. (2013) 14:646–53. 10.1038/ni.2604 23778791 PMC3845005

[56] Chu H Mazmanian SK. Innate immune recognition of the microbiota promotes host-microbial symbiosis. Nat Immunol. (2013) 14:668–75. 10.1038/ni.2635 23778794 PMC4109969

[57] Iida N Dzutsev A Stewart CA Smith L Bouladoux N Weingarten RA. Commensal bacteria control cancer response to therapy by modulating the tumor microenvironment. Science. (2013) 342:967–70. 10.1126/science.1240527 24264989 PMC6709532

[58] Lanzavecchia A Sallusto F. Regulation of T cell immunity by dendritic cells. Cell. (2001) 106:263–6. 10.1016/S0092-8674(01)00455-X 11509174

[59] Schulz-Kuhnt A Wirtz S Neurath MF Atreya I. Regulation of human innate lymphoid cells in the context of mucosal inflammation. Front Immunol. (2020) 11:1062. 10.3389/fimmu.2020.01062 32655549 PMC7324478

[60] Agrawal S Agrawal A Doughty B Gerwitz A Blenis J Van Dyke T. Cutting edge: different toll-like receptor agonists instruct dendritic cells to induce distinct Th responses via differential modulation of extracellular signal-regulated kinase-mitogen-activated protein kinase and c-Fos. J Immunol. (2003) 171:4984–9. 10.4049/jimmunol.171.10.4984 14607893

[61] Pasare C Medzhitov R. Toll-dependent control mechanisms of CD4 T cell activation. Immunity. (2004) 21:733–41. 10.1016/j.immuni.2004.10.006 15539158

[62] Pasare C Medzhitov R. Toll-like receptors and acquired immunity. Semin Immunol. (2004) 16:23–6. 10.1016/j.smim.2003.10.006 14751760

[63] Pasare C Medzhitov R. Toll-like receptors: linking innate and adaptive immunity. Microbes Infect. (2004) 6:1382–7. 10.1016/j.micinf.2004.08.018 15596124

[64] Kroll P Knoke K Steiger J Fabri M. IFN-gamma promotes, but dexamethasone dissociates, toll-like receptor 2/1-induced host responses in human macrophages. J Invest Dermatol. (2019) 139:488–91. 10.1016/j.jid.2018.07.035 30193757

[65] Lloyd CM Snelgrove RJ. Type 2 immunity: expanding our view. Sci Immunol. (2018) 3:eaat1604. 10.1126/sciimmunol.aat1604 29980619

[66] Gaffen SL Jain R Garg AV Cua DJ. The IL-23-IL-17 immune axis: from mechanisms to therapeutic testing. Nat Rev Immunol. (2014) 14:585–600. 10.1038/nri3707 25145755 PMC4281037

[67] Marks KE Flaherty S Patterson KM Stratton M Martinez GJ Reynolds JM. Toll-like receptor 2 induces pathogenicity in Th17 cells and reveals a role for IPCEF in regulating Th17 cell migration. Cell Rep. (2021) 35:109303. 10.1016/j.celrep.2021.109303 34192530 PMC8270556

[68] Van Maele L Carnoy C Cayet D Songhet P Dumoutier L Ferrero I. TLR5 signaling stimulates the innate production of IL-17 and IL-22 by CD3(neg)CD127+ immune cells in spleen and mucosa. J Immunol. (2010) 185:1177–85. 10.4049/jimmunol.1000115 20566828 PMC3060348

[69] Gottschalk RA Martins AJ Angermann BR Dutta B Ng CE Uderhardt S. Distinct NF-kappaB and MAPK activation thresholds uncouple steady-state microbe sensing from anti-pathogen inflammatory responses. Cell Syst. (2016) 2:378–90. 10.1016/j.cels.2016.04.016 27237739 PMC4919147

[70] Tian M Hua Z Hong S Zhang Z Liu C Lin L. B cell-intrinsic MyD88 signaling promotes initial cell proliferation and differentiation to enhance the germinal center response to a virus-like particle. J Immunol. (2018) 200:937–48. 10.4049/jimmunol.1701067 29282308

[71] Imanishi T Saito T. T cell co-stimulation and functional modulation by innate signals. Trends Immunol. (2020) 41:200–12. 10.1016/j.it.2020.01.003 32035763

[72] Barnes MJ Powrie F. Regulatory T cells reinforce intestinal homeostasis. Immunity. (2009) 31:401–11. 10.1016/j.immuni.2009.08.011 19766083

[73] Mantis NJ Rol N Corthesy B. Secretory IgA's complex roles in immunity and mucosal homeostasis in the gut. Mucosal Immunol. (2011) 4:603–11. 10.1038/mi.2011.41 21975936 PMC3774538

[74] Dutzan N Konkel JE Greenwell-Wild T Moutsopoulos NM. Characterization of the human immune cell network at the gingival barrier. Mucosal Immunol. (2016) 9:1163–72. 10.1038/mi.2015.136 26732676 PMC4820049

[75] Cardoso CR Garlet GP Crippa GE Rosa AL Junior WM Rossi MA. Evidence of the presence of T helper type 17 cells in chronic lesions of human periodontal disease. Oral Microbiol Immunol. (2009) 24:1–6. 10.1111/j.1399-302X.2008.00463.x 19121062

[76] Moutsopoulos NM Kling HM Angelov N Jin W Palmer RJ Nares S. *Porphyromonas gingivalis* promotes Th17 inducing pathways in chronic periodontitis. J Autoimmun. (2012) 39:294–303. 10.1016/j.jaut.2012.03.003 22560973 PMC3416947

[77] Price AE Shamardani K Lugo KA Deguine J Roberts AW Lee BL. A map of toll-like receptor expression in the intestinal epithelium reveals distinct spatial, cell type-specific, and temporal patterns. Immunity. (2018) 49:560–75.e6. 10.1016/j.immuni.2018.07.016 30170812 PMC6152941

[78] Kuo IH Carpenter-Mendini A Yoshida T McGirt LY Ivanov AI Barnes KC. Activation of epidermal toll-like receptor 2 enhances tight junction function: implications for atopic dermatitis and skin barrier repair. J Invest Dermatol. (2013) 133:988–98. 10.1038/jid.2012.437 23223142 PMC3600383

[79] Yuki T Yoshida H Akazawa Y Komiya A Sugiyama Y Inoue S. Activation of TLR2 enhances tight junction barrier in epidermal keratinocytes. J Immunol. (2011) 187:3230–7. 10.4049/jimmunol.1100058 21841130

[80] Ruffner MA Song L Maurer K Shi L Carroll MC Wang JX. Toll-like receptor 2 stimulation augments esophageal barrier integrity. Allergy. (2019) 74:2449–60. 10.1111/all.13968 31267532 PMC7083217

[81] Hormann N Brandao I Jackel S Ens N Lillich M Walter U. Gut microbial colonization orchestrates TLR2 expression, signaling and epithelial proliferation in the small intestinal mucosa. PLoS ONE. (2014) 9:e113080. 10.1371/journal.pone.0113080 25396415 PMC4232598

[82] Cario E. Barrier-protective function of intestinal epithelial toll-like receptor 2. Mucosal Immunol. (2008) 1(Suppl. 1):S62–6. 10.1038/mi.2008.47 19079234

[83] Cario E Gerken G Podolsky DK. Toll-like receptor 2 controls mucosal inflammation by regulating epithelial barrier function. Gastroenterology. (2007) 132:1359–74. 10.1053/j.gastro.2007.02.056 17408640

[84] Pivarcsi A Bodai L Rethi B Kenderessy-Szabo A Koreck A Szell M. Expression and function of toll-like receptors 2 and 4 in human keratinocytes. Int Immunol. (2003) 15:721–30. 10.1093/intimm/dxg068 12750356

[85] Goleva E Berdyshev E Leung DY. Epithelial barrier repair and prevention of allergy. J Clin Invest. (2019) 129:1463–74. 10.1172/JCI124608 30776025 PMC6436854

[86] Mullin JM Snock KV. Effect of tumor necrosis factor on epithelial tight junctions and transepithelial permeability. Cancer Res. (1990) 50:2172–6. 2180562

[87] Schauber J Dorschner RA Coda AB Buchau AS Liu PT Kiken D. Injury enhances TLR2 function and antimicrobial peptide expression through a vitamin D-dependent mechanism. J Clin Invest. (2007) 117:803–11. 10.1172/JCI30142 17290304 PMC1784003

[88] Schauber J Gallo RL. Expanding the roles of antimicrobial peptides in skin: alarming and arming keratinocytes. J Invest Dermatol. (2007) 127:510–2. 10.1038/sj.jid.5700761 17299432

[89] Scheeren FA Kuo AH van Weele LJ Cai S Glykofridis I Sikandar SS. A cell-intrinsic role for TLR2-MYD88 in intestinal and breast epithelia and oncogenesis. Nat Cell Biol. (2014) 16:1238–48. 10.1038/ncb3058 25362351

[90] Neal MD Sodhi CP Jia H Dyer M Egan CE Yazji I. Toll-like receptor 4 is expressed on intestinal stem cells and regulates their proliferation and apoptosis via the p53 up-regulated modulator of apoptosis. J Biol Chem. (2012) 287:37296–308. 10.1074/jbc.M112.375881 22955282 PMC3481327

[91] Koh J Kurago ZB. Expanded expression of toll-like receptor 2 in proliferative verrucous leukoplakia. Head Neck Pathol. (2019) 13:635–42. 10.1007/s12105-019-01028-y 30888638 PMC6854203

[92] Beklen A Hukkanen M Richardson R Konttinen YT. Immunohistochemical localization of toll-like receptors 1-10 in periodontitis. Oral Microbiol Immunol. (2008) 23:425–31. 10.1111/j.1399-302X.2008.00448.x 18793367

[93] Beklen A Sorsa T Konttinen YT. Toll-like receptors 2 and 5 in human gingival epithelial cells co-operate with T-cell cytokine interleukin-17. Oral Microbiol Immunol. (2009) 24:38–42. 10.1111/j.1399-302X.2008.00473.x 19121068

[94] Guo W Wang P Liu ZH Ye P. Analysis of differential expression of tight junction proteins in cultured oral epithelial cells altered by *Porphyromonas gingivalis, Porphyromonas gingivalis* lipopolysaccharide, and extracellular adenosine triphosphate. Int J Oral Sci. (2018) 10:e8. 10.1038/ijos.2017.51 29319048 PMC5795020

[95] McClure R Massari P. TLR-dependent human mucosal epithelial cell responses to microbial pathogens. Front Immunol. (2014) 5:386. 10.3389/fimmu.2014.00386 25161655 PMC4129373

[96] Palani CD Ramanathapuram L Lam-Ubol A Kurago ZB. Toll-like receptor 2 induces adenosine receptor A2a and promotes human squamous carcinoma cell growth via extracellular signal regulated kinases (1/2). Oncotarget. (2018) 9:6814–29. 10.18632/oncotarget.23784 29467931 PMC5805517

[97] Sugawara Y Uehara A Fujimoto Y Kusumoto S Fukase K Shibata K. Toll-like receptors, NOD1, and NOD2 in oral epithelial cells. J Dent Res. (2006) 85:524–9. 10.1177/154405910608500609 16723649

[98] Uehara A Fujimoto Y Fukase K Takada H. Various human epithelial cells express functional Toll-like receptors, NOD1 and NOD2 to produce anti-microbial peptides, but not proinflammatory cytokines. Mol Immunol. (2007) 44:3100–11. 10.1016/j.molimm.2007.02.007 17403538

[99] Uehara A Sugawara S Takada H. Priming of human oral epithelial cells by interferon-gamma to secrete cytokines in response to lipopolysaccharides, lipoteichoic acids and peptidoglycans. J Med Microbiol. (2002) 51:626–34. 10.1099/0022-1317-51-8-626 12171292

[100] Chung WO Dale BA. Differential utilization of nuclear factor-kappaB signaling pathways for gingival epithelial cell responses to oral commensal and pathogenic bacteria. Oral Microbiol Immunol. (2008) 23:119–26. 10.1111/j.1399-302X.2007.00398.x 18279179 PMC2826319

[101] Kantrong N To TT Darveau RP. Gingival epithelial cell recognition of lipopolysaccharide. Adv Exp Med Biol. (2019) 1197:55–67. 10.1007/978-3-030-28524-1_5 31732934

[102] Omar AA Korvala J Haglund C Virolainen S Hayry V Atula T. Toll-like receptors−4 and−5 in oral and cutaneous squamous cell carcinomas. J Oral Pathol Med. (2015) 44:258–65. 10.1111/jop.12233 25047824

[103] Kauppila JH Mattila AE Karttunen TJ Salo T. Toll-like receptor 5 (TLR5) expression is a novel predictive marker for recurrence and survival in squamous cell carcinoma of the tongue. Br J Cancer. (2013) 108:638–43. 10.1038/bjc.2012.589 23287987 PMC3593548

[104] Helminen O Huhta H Takala H Lehenkari PP Saarnio J Kauppila JH. Increased toll-like receptor 5 expression indicates esophageal columnar dysplasia. Virchows Arch. (2014) 464:11–8. 10.1007/s00428-013-1505-2 24221343

[105] Pimentel-Nunes P Afonso L Lopes P Roncon-Albuquerque R Jr Goncalves N Henrique R. Increased expression of toll-like receptors (TLR) 2, 4 and 5 in gastric dysplasia. Pathol Oncol Res. (2011) 17:677–83. 10.1007/s12253-011-9368-9 21455638

[106] Burgueno JF Fritsch J Gonzalez EE Landau KS Santander AM Fernandez I. Epithelial TLR4 signaling activates DUOX2 to induce microbiota-driven tumorigenesis. Gastroenterology. (2021) 160:797–808.e6. 10.1053/j.gastro.2020.10.031 33127391 PMC7879481

[107] Farnebo L Shahangian A Lee Y Shin JH Scheeren FA Sunwoo JB. Targeting Toll-like receptor 2 inhibits growth of head and neck squamous cell carcinoma. Oncotarget. (2015) 6:9897–907. 10.18632/oncotarget.3393 25846753 PMC4496405

[108] Ikehata N Takanashi M Satomi T Watanabe M Hasegawa O Kono M. Toll-like receptor 2 activation implicated in oral squamous cell carcinoma development. Biochem Biophys Res Commun. (2018) 495:2227–34. 10.1016/j.bbrc.2017.12.098 29269299

[109] Kurago ZB Lam-ubol A Stetsenko A De La Mater C Chen Y Dawson DV. Lipopolysaccharide-squamous cell carcinoma-monocyte interactions induce cancer-supporting factors leading to rapid STAT3 activation. Head Neck Pathol. (2008) 2:1–12. 10.1007/s12105-007-0038-x 19603082 PMC2709294

[110] Szczepanski MJ Czystowska M Szajnik M Harasymczuk M Boyiadzis M Kruk-Zagajewska A. Triggering of toll-like receptor 4 expressed on human head and neck squamous cell carcinoma promotes tumor development and protects the tumor from immune attack. Cancer Res. (2009) 69:3105–13. 10.1158/0008-5472.CAN-08-3838 19318560 PMC3708458

[111] Kratochvill F Neale G Haverkamp JM Van de Velde LA Smith AM Kawauchi D. TNF counterbalances the emergence of M2 tumor macrophages. Cell Rep. (2015) 12:1902–14. 10.1016/j.celrep.2015.08.033 26365184 PMC4581986

[112] Grivennikov S Karin E Terzic J Mucida D Yu GY Vallabhapurapu S. IL-6 and Stat3 are required for survival of intestinal epithelial cells and development of colitis-associated cancer. Cancer Cell. (2009) 15:103–13. 10.1016/j.ccr.2009.01.001 19185845 PMC2667107

[113] Kuhn KA Manieri NA Liu TC Stappenbeck TS. IL-6 stimulates intestinal epithelial proliferation and repair after injury. PLoS ONE. (2014) 9:e114195. 10.1371/journal.pone.0114195 25478789 PMC4257684

[114] Taniguchi K Karin M. IL-6 and related cytokines as the critical lynchpins between inflammation and cancer. Semin Immunol. (2014) 26:54–74. 10.1016/j.smim.2014.01.001 24552665

[115] Saraiva M O'Garra A. The regulation of IL-10 production by immune cells. Nat Rev Immunol. (2010) 10:170–81. 10.1038/nri2711 20154735

[116] Lampropoulou V Hoehlig K Roch T Neves P Calderon Gomez E Sweenie CH. TLR-activated B cells suppress T cell-mediated autoimmunity. J Immunol. (2008) 180:4763–73. 10.4049/jimmunol.180.7.4763 18354200

[117] Hayes CL Dong J Galipeau HJ Jury J McCarville J Huang X. Commensal microbiota induces colonic barrier structure and functions that contribute to homeostasis. Sci Rep. (2018) 8:14184. 10.1038/s41598-018-32366-6 30242285 PMC6155058

[118] Hamidzadeh K Mosser DM. Purinergic signaling to terminate TLR responses in macrophages. Front Immunol. (2016) 7:74. 10.3389/fimmu.2016.00074 26973651 PMC4773587

[119] Cohen HB Briggs KT Marino JP Ravid K Robson SC Mosser DM. TLR stimulation initiates a CD39-based autoregulatory mechanism that limits macrophage inflammatory responses. Blood. (2013) 122:1935–45. 10.1182/blood-2013-04-496216 23908469 PMC3772500

[120] Koga K Takaesu G Yoshida R Nakaya M Kobayashi T Kinjyo I. Cyclic adenosine monophosphate suppresses the transcription of proinflammatory cytokines via the phosphorylated c-Fos protein. Immunity. (2009) 30:372–83. 10.1016/j.immuni.2008.12.021 19285436

[121] Kreckler LM Gizewski E Wan TC Auchampach JA. Adenosine suppresses lipopolysaccharide-induced tumor necrosis factor-alpha production by murine macrophages through a protein kinase A- and exchange protein activated by cAMP-independent signaling pathway. J Pharmacol Exp Ther. (2009) 331:1051–61. 10.1124/jpet.109.157651 19749080 PMC2784717

[122] Kreckler LM Wan TC Ge ZD Auchampach JA. Adenosine inhibits tumor necrosis factor-alpha release from mouse peritoneal macrophages via A2A and A2B but not the A3 adenosine receptor. J Pharmacol Exp Ther. (2006) 317:172–80. 10.1124/jpet.105.096016 16339914

[123] Clark IA. How TNF was recognized as a key mechanism of disease. Cytokine Growth Factor Rev. (2007) 18:335–43. 10.1016/j.cytogfr.2007.04.002 17493863

[124] Puchta A Naidoo A Verschoor CP Loukov D Thevaranjan N Mandur TS. TNF drives monocyte dysfunction with age and results in impaired anti-pneumococcal immunity. PLoS Pathog. (2016) 12:e1005368. 10.1371/journal.ppat.1005368 26766566 PMC4713203

[125] Roca FJ Ramakrishnan L. TNF dually mediates resistance and susceptibility to mycobacteria via mitochondrial reactive oxygen species. Cell. (2013) 153:521–34. 10.1016/j.cell.2013.03.022 23582643 PMC3790588

[126] Hoving S Seynhaeve AL van Tiel ST aan de Wiel-Ambagtsheer G de Bruijn EA Eggermont AM ten Hagen TL. Early destruction of tumor vasculature in tumor necrosis factor-alpha-based isolated limb perfusion is responsible for tumor response. Anticancer Drugs. (2006) 17:949–59. 10.1097/01.cad.0000224450.54447.b3 16940805

[127] Josephs SF Ichim TE Prince SM Kesari S Marincola FM Escobedo AR. Unleashing endogenous TNF-alpha as a cancer immunotherapeutic. J Transl Med. (2018) 16:242. 10.1186/s12967-018-1611-7 30170620 PMC6119315

[128] Young A Mittal D Stagg J Smyth MJ. Targeting cancer-derived adenosine: new therapeutic approaches. Cancer Discov. (2014) 4:879–88. 10.1158/2159-8290.CD-14-0341 25035124

[129] Montesinos MC Desai-Merchant A Cronstein BN. Promotion of wound healing by an agonist of adenosine A2A receptor is dependent on tissue plasminogen activator. Inflammation. (2015) 38:2036–41. 10.1007/s10753-015-0184-3 25991438

[130] Murray PJ Wynn TA. Protective and pathogenic functions of macrophage subsets. Nat Rev Immunol. (2011) 11:723–37. 10.1038/nri3073 21997792 PMC3422549

[131] Rius J Guma M Schachtrup C Akassoglou K Zinkernagel AS Nizet V. NF-kappaB links innate immunity to the hypoxic response through transcriptional regulation of HIF-1alpha. Nature. (2008) 453:807–11. 10.1038/nature06905 18432192 PMC2669289

[132] Lavoie H Gagnon J Therrien M. ERK signalling: a master regulator of cell behaviour, life and fate. Nat Rev Mol Cell Biol. (2020) 21:607–32. 10.1038/s41580-020-0255-7 32576977

[133] Sequeira I Neves JF Carrero D Peng Q Palasz N Liakath-Ali K. Immunomodulatory role of Keratin 76 in oral and gastric cancer. Nat Commun. (2018) 9:3437. 10.1038/s41467-018-05872-4 30143634 PMC6109110

[134] Helminen O Huhta H Leppanen J Kauppila JH Takala H Lehenkari PP. Nuclear localization of Toll-like receptor 5 in Barrett's esophagus and esophageal adenocarcinoma is associated with metastatic behavior. Virchows Arch. (2016) 469:465–70. 10.1007/s00428-016-1989-7 27392931

[135] Huhta H Helminen O Lehenkari PP Saarnio J Karttunen TJ Kauppila JH. Toll-like receptors 1, 2, 4 and 6 in esophageal epithelium, Barrett's esophagus, dysplasia and adenocarcinoma. Oncotarget. (2016) 7:23658–67. 10.18632/oncotarget.8151 27008696 PMC5029654

[136] Kim WY Lee JW Choi JJ Choi CH Kim TJ Kim BG. Increased expression of toll-like receptor 5 during progression of cervical neoplasia. Int J Gynecol Cancer. (2008) 18:300–5. 10.1111/j.1525-1438.2007.01008.x 17587322

[137] Santaolalla R Sussman DA Ruiz JR Davies JM Pastorini C Espana CL. TLR4 activates the beta-catenin pathway to cause intestinal neoplasia. PLoS ONE. (2013) 8:e63298. 10.1371/journal.pone.0063298 23691015 PMC3653932

[138] Grivennikov SI Wang K Mucida D Stewart CA Schnabl B Jauch D. Adenoma-linked barrier defects and microbial products drive IL-23/IL-17-mediated tumour growth. Nature. (2012) 491:254–8. 10.1038/nature11465 23034650 PMC3601659

[139] McGeachy MJ Cua DJ Gaffen SL. The IL-17 Family of cytokines in health and disease. Immunity. (2019) 50:892–906. 10.1016/j.immuni.2019.03.021 30995505 PMC6474359

[140] Johnson SD De Costa AM Young MR. Effect of the premalignant and tumor microenvironment on immune cell cytokine production in head and neck cancer. Cancers. (2014) 6:756–70. 10.3390/cancers6020756 24698959 PMC4074802

[141] Woodford D Johnson SD De Costa AM Young MR. An inflammatory cytokine milieu is prominent in premalignant oral lesions, but subsides when lesions progress to squamous cell carcinoma. J Clin Cell Immunol. (2014) 5:3. 10.4172/2155-9899.1000230 25419481 PMC4240319

[142] Caughron B Yang Y Young MRI. Role of IL-23 signaling in the progression of premalignant oral lesions to cancer. PLoS ONE. (2018) 13:e0196034. 10.1371/journal.pone.0196034 29664967 PMC5903614

[143] Foy JP Bertolus C Ortiz-Cuaran S Albaret MA Williams WN Lang W. Immunological and classical subtypes of oral premalignant lesions. Oncoimmunology. (2018) 7:e1496880. 10.1080/2162402X.2018.1496880 30524889 PMC6279331

[144] Li B Cui Y Nambiar DK Sunwoo JB Li R. The immune subtypes and landscape of squamous cell carcinoma. Clin Cancer Res. (2019) 25:3528–37. 10.1158/1078-0432.CCR-18-4085 30833271 PMC6571041

[145] Cillo AR Kurten CHL Tabib T Qi Z Onkar S Wang T. Immune landscape of viral- and carcinogen-driven head and neck cancer. Immunity. (2020) 52:183–99.e9. 10.1016/j.immuni.2019.11.014 31924475 PMC7201194

[146] Barros LRC Souza-Santos PT Pretti MAM Vieira GF Bragatte MAS Mendes MFA. High infiltration of B cells in tertiary lymphoid structures, TCR oligoclonality, and neoantigens are part of esophageal squamous cell carcinoma microenvironment. J Leukoc Biol. (2020) 108:1307–18. 10.1002/JLB.5MA0720-710RRR 32827331

[147] Gago da Graca C van Baarsen LGM Mebius RE. Tertiary lymphoid structures: diversity in their development, composition, and role. J Immunol. (2021) 206:273–81. 10.4049/jimmunol.2000873 33397741

[148] Sautes-Fridman C Petitprez F Calderaro J Fridman WH. Tertiary lymphoid structures in the era of cancer immunotherapy. Nat Rev Cancer. (2019) 19:307–25. 10.1038/s41568-019-0144-6 31092904

[149] Sharonov GV Serebrovskaya EO Yuzhakova DV Britanova OV Chudakov DM. B cells, plasma cells and antibody repertoires in the tumour microenvironment. Nat Rev Immunol. (2020) 20:294–307. 10.1038/s41577-019-0257-x 31988391

[150] Wirsing AM Rikardsen OG Steigen SE Uhlin-Hansen L Hadler-Olsen E. Characterisation and prognostic value of tertiary lymphoid structures in oral squamous cell carcinoma. BMC Clin Pathol. (2014) 14:38. 10.1186/1472-6890-14-38 25177210 PMC4148494

[151] Dieu-Nosjean MC Goc J Giraldo NA Sautes-Fridman C Fridman WH. Tertiary lymphoid structures in cancer and beyond. Trends Immunol. (2014) 35:571–80. 10.1016/j.it.2014.09.006 25443495

[152] Brahmer JR. PD-1-targeted immunotherapy: recent clinical findings. Clin Adv Hematol Oncol. (2012) 10:674–5. 23187774

[153] Burtness B Haddad R Dinis J Trigo J Yokota T de Souza Viana L. Afatinib vs placebo as adjuvant therapy after chemoradiotherapy in squamous cell carcinoma of the head and neck: a randomized clinical trial. JAMA Oncol. (2019) 5:1170–80. 10.1001/jamaoncol.2019.1146 31194247 PMC6567846

[154] Byun DJ Wolchok JD Rosenberg LM Girotra M. Cancer immunotherapy - immune checkpoint blockade and associated endocrinopathies. Nat Rev Endocrinol. (2017) 13:195–207. 10.1038/nrendo.2016.205 28106152 PMC5629093

[155] Ferris RL Lenz HJ Trotta AM Garcia-Foncillas J Schulten J Audhuy F. Rationale for combination of therapeutic antibodies targeting tumor cells and immune checkpoint receptors: harnessing innate and adaptive immunity through IgG1 isotype immune effector stimulation. Cancer Treat Rev. (2018) 63:48–60. 10.1016/j.ctrv.2017.11.008 29223828 PMC7505164

[156] Ferriss JS Williams-Brown MY. Immunotherapy: checkpoint inhibitors in lynch-associated gynecologic cancers. Curr Treat Options Oncol. (2019) 20:75. 10.1007/s11864-019-0676-8 31444655

[157] Finn OJ. Human tumor antigens yesterday, today, and tomorrow. Cancer Immunol Res. (2017) 5:347–54. 10.1158/2326-6066.CIR-17-0112 28465452 PMC5490447

[158] Meeusen E Lim E Mathivanan S. Secreted tumor antigens - immune biomarkers for diagnosis and therapy. Proteomics. (2017) 17:1600442. 10.1002/pmic.201600442 28714192

[159] Scheper W Kelderman S Fanchi LF Linnemann C Bendle G de Rooij MAJ. Low and variable tumor reactivity of the intratumoral TCR repertoire in human cancers. Nat Med. (2019) 25:89–94. 10.1038/s41591-018-0266-5 30510250

[160] Kimura T McKolanis JR Dzubinski LA Islam K Potter DM Salazar AM. MUC1 vaccine for individuals with advanced adenoma of the colon: a cancer immunoprevention feasibility study. Cancer Prev Res. (2013) 6:18–26. 10.1158/1940-6207.CAPR-12-0275 23248097 PMC3536916

[161] Greene S Robbins Y Mydlarz WK Huynh AP Schmitt NC Friedman J. Inhibition of MDSC trafficking with SX-682, a CXCR1/2 inhibitor, enhances NK-cell immunotherapy in head and neck cancer models. Clin Cancer Res. (2020) 26:1420–31. 10.1158/1078-0432.CCR-19-2625 31848188 PMC7073293

[162] Yeh DW Chen YS Lai CY Liu YL Lu CH Lo JF. Downregulation of COMMD1 by miR-205 promotes a positive feedback loop for amplifying inflammatory- and stemness-associated properties of cancer cells. Cell Death Differ. (2016) 23:841–52. 10.1038/cdd.2015.147 26586569 PMC4832103

[163] Rutkowski MR Conejo-Garcia JR. TLR5 signaling, commensal microbiota and systemic tumor promoting inflammation: the three parcae of malignant progression. Oncoimmunology. (2015) 4:e1021542. 10.1080/2162402X.2015.1021542 26405577 PMC4570098

[164] Laviron M Combadiere C Boissonnas A. Tracking monocytes and macrophages in tumors with live imaging. Front Immunol. (2019) 10:1201. 10.3389/fimmu.2019.01201 31214174 PMC6555099

[165] Hughes CE Nibbs RJB. A guide to chemokines and their receptors. FEBS J. (2018) 285:2944–71. 10.1111/febs.14466 29637711 PMC6120486

[166] Panda S Padhiary SK Routray S. Chemokines accentuating protumoral activities in oral cancer microenvironment possess an imperious stratagem for therapeutic resolutions. Oral Oncol. (2016) 60:8–17. 10.1016/j.oraloncology.2016.06.008 27531867

[167] Ghosh TK Mickelson DJ Fink J Solberg JC Inglefield JR Hook D. Toll-like receptor (TLR) 2-9 agonists-induced cytokines and chemokines: I. Comparison with T cell receptor-induced responses. Cell Immunol. (2006) 243:48–57. 10.1016/j.cellimm.2006.12.002 17250816

[168] Re F Strominger JL. Toll-like receptor 2 (TLR2) and TLR4 differentially activate human dendritic cells. J Biol Chem. (2001) 276:37692–9. 10.1074/jbc.M105927200 11477091

[169] Taniguchi K Karin M. NF-kappaB, inflammation, immunity and cancer: coming of age. Nat Rev Immunol. (2018) 18:309–24. 10.1038/nri.2017.142 29379212

[170] Vitale I Manic G Coussens LM Kroemer G Galluzzi L. Macrophages and metabolism in the tumor microenvironment. Cell Metab. (2019) 30:36–50. 10.1016/j.cmet.2019.06.001 31269428

[171] Veglia F Sanseviero E Gabrilovich DI. Myeloid-derived suppressor cells in the era of increasing myeloid cell diversity. Nat Rev Immunol. (2021) 21:485–98. 10.1038/s41577-020-00490-y 33526920 PMC7849958

[172] Shime H Maruyama A Yoshida S Takeda Y Matsumoto M Seya T. Toll-like receptor 2 ligand and interferon-gamma suppress anti-tumor T cell responses by enhancing the immunosuppressive activity of monocytic myeloid-derived suppressor cells. Oncoimmunology. (2017) 7:e1373231. 10.1080/2162402X.2017.1373231 29296526 PMC5739553

[173] Lauterbach MA Hanke JE Serefidou M Mangan MSJ Kolbe CC Hess T. Toll-like receptor signaling rewires macrophage metabolism and promotes histone acetylation via ATP-citrate lyase. Immunity. (2019) 51:997–1011.e7. 10.1016/j.immuni.2019.11.009 31851905

[174] Nonnenmacher Y Hiller K. Biochemistry of proinflammatory macrophage activation. Cell Mol Life Sci. (2018) 75:2093–109. 10.1007/s00018-018-2784-1 29502308 PMC5948278

[175] Guilliams M Thierry GR Bonnardel J Bajenoff M. Establishment and maintenance of the macrophage niche. Immunity. (2020) 52:434–51. 10.1016/j.immuni.2020.02.015 32187515

[176] Cassetta L Kitamura T. Macrophage targeting: opening new possibilities for cancer immunotherapy. Immunology. (2018) 155:285–93. 10.1111/imm.12976 29963704 PMC6187207

[177] Coussens LM Zitvogel L Palucka AK. Neutralizing tumor-promoting chronic inflammation: a magic bullet? Science. (2013) 339:286–91. 10.1126/science.1232227 23329041 PMC3591506

[178] Mantovani A Marchesi F Malesci A Laghi L Allavena P. Tumour-associated macrophages as treatment targets in oncology. Nat Rev Clin Oncol. (2017) 14:399–416. 10.1038/nrclinonc.2016.217 28117416 PMC5480600

[179] Lam-ubol A Hopkin D Letuchy EM Kurago ZB. Squamous carcinoma cells influence monocyte phenotype and suppress lipopolysaccharide-induced TNF-alpha in monocytes. Inflammation. (2010) 33:207–23. 10.1007/s10753-009-9175-6 20084448 PMC2888725

[180] Rajarathnam K Schnoor M Richardson RM Rajagopal S. How do chemokines navigate neutrophils to the target site: dissecting the structural mechanisms and signaling pathways. Cell Signal. (2019) 54:69–80. 10.1016/j.cellsig.2018.11.004 30465827 PMC6664297

[181] Shinriki S Jono H Ueda M Ota K Ota T Sueyoshi T. Interleukin-6 signalling regulates vascular endothelial growth factor-C synthesis and lymphangiogenesis in human oral squamous cell carcinoma. J Pathol. (2011) 225:142–50. 10.1002/path.2935 21710490

[182] Blaser H Dostert C Mak TW Brenner D. TNF and ROS crosstalk in inflammation. Trends Cell Biol. (2016) 26:249–61. 10.1016/j.tcb.2015.12.002 26791157

[183] Balkwill F. Tumor necrosis factor or tumor promoting factor? Cytokine Growth Factor Rev. (2002) 13:135–41. 10.1016/S1359-6101(01)00020-X 11900989

[184] Salomon BL Leclerc M Tosello J Ronin E Piaggio E Cohen JL. Tumor necrosis factor alpha and regulatory T cells in oncoimmunology. Front Immunol. (2018) 9:444. 10.3389/fimmu.2018.00444 29593717 PMC5857565

[185] Allard D Turcotte M Stagg J. Targeting A2 adenosine receptors in cancer. Immunol Cell Biol. (2017) 95:333–9. 10.1038/icb.2017.8 28174424

[186] Vigano S Alatzoglou D Irving M Menetrier-Caux C Caux C Romero P. Targeting adenosine in cancer immunotherapy to enhance T-cell function. Front Immunol. (2019) 10:925. 10.3389/fimmu.2019.00925 31244820 PMC6562565

[187] Mempin R Tran H Chen C Gong H Kim Ho K Lu S. Release of extracellular ATP by bacteria during growth. BMC Microbiol. (2013) 13:301. 10.1186/1471-2180-13-301 24364860 PMC3882102

[188] Blay J White TD Hoskin DW. The extracellular fluid of solid carcinomas contains immunosuppressive concentrations of adenosine. Cancer Res. (1997) 57:2602–5. 9205063

[189] Narasimhan PB Marcovecchio P Hamers AAJ Hedrick CC. Nonclassical monocytes in health and disease. Annu Rev Immunol. (2019) 37:439–56. 10.1146/annurev-immunol-042617-053119 31026415

[190] Kasama H Sakamoto Y Kasamatsu A Okamoto A Koyama T Minakawa Y. Adenosine A2b receptor promotes progression of human oral cancer. BMC Cancer. (2015) 15:563. 10.1186/s12885-015-1577-2 26228921 PMC4520274

[191] Grivennikov SI Karin M. Inflammatory cytokines in cancer: tumour necrosis factor and interleukin 6 take the stage. Ann Rheum Dis. (2011) 70(Suppl. 1):i104–8. 10.1136/ard.2010.140145 21339211

[192] Hunter KD Parkinson EK Harrison PR. Profiling early head and neck cancer. Nat Rev Cancer. (2005) 5:127–35. 10.1038/nrc1549 15685196

[193] Yu H Lee H Herrmann A Buettner R Jove R. Revisiting STAT3 signalling in cancer: new and unexpected biological functions. Nat Rev Cancer. (2014) 14:736–46. 10.1038/nrc3818 25342631

[194] Tang M Diao J Gu H Khatri I Zhao J Cattral MS. Toll-like receptor 2 activation promotes tumor dendritic cell dysfunction by regulating IL-6 and IL-10 receptor signaling. Cell Rep. (2015) 13:2851–64. 10.1016/j.celrep.2015.11.053 26711349

[195] Koltsova EK Grivennikov SI. IL-22 gets to the stem of colorectal cancer. Immunity. (2014) 40:639–41. 10.1016/j.immuni.2014.04.014 24837100

[196] Quesnelle KM Boehm AL Grandis JR. STAT-mediated EGFR signaling in cancer. J Cell Biochem. (2007) 102:311–9. 10.1002/jcb.21475 17661350

[197] Haque A Moriyama M Kubota K Ishiguro N Sakamoto M Chinju A. CD206(+) tumor-associated macrophages promote proliferation and invasion in oral squamous cell carcinoma via EGF production. Sci Rep. (2019) 9:14611. 10.1038/s41598-019-51149-1 31601953 PMC6787225

[198] Niu G Wright KL Huang M Song L Haura E Turkson J. Constitutive Stat3 activity up-regulates VEGF expression and tumor angiogenesis. Oncogene. (2002) 21:2000–8. 10.1038/sj.onc.1205260 11960372

[199] Xi S Gooding WE Grandis JR. *In vivo* antitumor efficacy of STAT3 blockade using a transcription factor decoy approach: implications for cancer therapy. Oncogene. (2005) 24:970–9. 10.1038/sj.onc.1208316 15592503

[200] Gentles AJ Newman AM Liu CL Bratman SV Feng W Kim D. The prognostic landscape of genes and infiltrating immune cells across human cancers. Nat Med. (2015) 21:938–45. 10.1038/nm.3909 26193342 PMC4852857

[201] Caldeira PC Vieira ELM Sousa AA Teixeira AL Aguiar MCF. Immunophenotype of neutrophils in oral squamous cell carcinoma patients. J Oral Pathol Med. (2017) 46:703–9. 10.1111/jop.12575 28370402

[202] Magalhaes MA Glogauer JE Glogauer M. Neutrophils and oral squamous cell carcinoma: lessons learned and future directions. J Leukoc Biol. (2014) 96:695–702. 10.1189/jlb.4RU0614-294R 25145471

[203] Perisanidis C Kornek G Poschl PW Holzinger D Pirklbauer K Schopper C. High neutrophil-to-lymphocyte ratio is an independent marker of poor disease-specific survival in patients with oral cancer. Med Oncol. (2013) 30:334. 10.1007/s12032-012-0334-5 23292862

[204] Kurt-Jones EA Mandell L Whitney C Padgett A Gosselin K Newburger PE. Role of toll-like receptor 2 (TLR2) in neutrophil activation: GM-CSF enhances TLR2 expression and TLR2-mediated interleukin 8 responses in neutrophils. Blood. (2002) 100:1860–8. 10.1182/blood.V100.5.1860.h81702001860_1860_1868 12176910

[205] Prince LR Whyte MK Sabroe I Parker LC. The role of TLRs in neutrophil activation. Curr Opin Pharmacol. (2011) 11:397–403. 10.1016/j.coph.2011.06.007 21741310

[206] Sabroe I Prince LR Jones EC Horsburgh MJ Foster SJ Vogel SN. Selective roles for Toll-like receptor (TLR)2 and TLR4 in the regulation of neutrophil activation and life span. J Immunol. (2003) 170:5268–75. 10.4049/jimmunol.170.10.5268 12734376

[207] Zhou J Nefedova Y Lei A Gabrilovich D. Neutrophils and PMN-MDSC: their biological role and interaction with stromal cells. Semin Immunol. (2018) 35:19–28. 10.1016/j.smim.2017.12.004 29254756 PMC5866202

[208] Slater TW Finkielsztein A Mascarenhas LA Mehl LC Butin-Israeli V Sumagin R. Neutrophil microparticles deliver active myeloperoxidase to injured mucosa to inhibit epithelial wound healing. J Immunol. (2017) 198:2886–97. 10.4049/jimmunol.1601810 28242649 PMC5360559

[209] Lee JJ Kao KC Chiu YL Jung CJ Liu CJ Cheng SJ. Enrichment of human CCR6(+) regulatory T cells with superior suppressive activity in oral cancer. J Immunol. (2017) 199:467–76. 10.4049/jimmunol.1601815 28600287

[210] Liu JY Li F Wang LP Chen XF Wang D Cao L. CTL- vs Treg lymphocyte-attracting chemokines, CCL4 and CCL20, are strong reciprocal predictive markers for survival of patients with oesophageal squamous cell carcinoma. Br J Cancer. (2015) 113:747–55. 10.1038/bjc.2015.290 26284335 PMC4559838

[211] Wirsing AM Ervik IK Seppola M Uhlin-Hansen L Steigen SE Hadler-Olsen E. Presence of high-endothelial venules correlates with a favorable immune microenvironment in oral squamous cell carcinoma. Mod Pathol. (2018) 31:910–22. 10.1038/s41379-018-0019-5 29416107

[212] Ueda S Goto M Hashimoto K Hasegawa S Imazawa M Takahashi M. Salivary CCL20 level as a biomarker for oral squamous cell carcinoma. Cancer Genomics Proteomics. (2021) 18:103–12. 10.21873/cgp.20245 33608307 PMC7943213

[213] Yamazaki S Okada K Maruyama A Matsumoto M Yagita H Seya T. TLR2-dependent induction of IL-10 and Foxp3+ CD25+ CD4+ regulatory T cells prevents effective anti-tumor immunity induced by Pam2 lipopeptides *in vivo*. PLoS ONE. (2011) 6:e18833. 10.1371/journal.pone.0018833 21533081 PMC3080372

[214] Boxberg M Leising L Steiger K Jesinghaus M Alkhamas A Mielke M. Composition and clinical impact of the immunologic tumor microenvironment in oral squamous cell carcinoma. J Immunol. (2019) 202:278–91. 10.4049/jimmunol.1800242 30530592

[215] Fridman WH. The immune microenvironment as a guide for cancer therapies. Oncoimmunology. (2012) 1:261–2. 10.4161/onci.19651 22737600 PMC3382864

[216] Lee AYS Korner H. The CCR6-CCL20 axis in humoral immunity and T-B cell immunobiology. Immunobiology. (2019) 224:449–54. 10.1016/j.imbio.2019.01.005 30772094

[217] Salvador B Arranz A Francisco S Cordoba L Punzon C Llamas MA. Modulation of endothelial function by toll like receptors. Pharmacol Res. (2016) 108:46–56. 10.1016/j.phrs.2016.03.038 27073018

[218] Koliaraki V Chalkidi N Henriques A Tzaferis C Polykratis A Waisman A. Innate sensing through mesenchymal TLR4/MyD88 signals promotes spontaneous intestinal tumorigenesis. Cell Rep. (2019) 26:536–45.e4. 10.1016/j.celrep.2018.12.072 30650348 PMC6334226

[219] Frank M Hennenberg EM Eyking A Runzi M Gerken G Scott P. TLR signaling modulates side effects of anticancer therapy in the small intestine. J Immunol. (2015) 194:1983–95. 10.4049/jimmunol.1402481 25589072 PMC4338614

[220] Jouhi L Mohamed H Makitie A Remes SM Haglund C Atula T. Toll-like receptor 5 and 7 expression may impact prognosis of HPV-positive oropharyngeal squamous cell carcinoma patients. Cancer Immunol Immunother. (2017) 66:1619–29. 10.1007/s00262-017-2054-3 28856441 PMC11028863

[221] Kylma AK Tolvanen TA Carpen T Haglund C Makitie A Mattila PS. Elevated TLR5 expression *in vivo* and loss of NF-kappaBeta activation via TLR5 *in vitro* detected in HPV-negative oropharyngeal squamous cell carcinoma. Exp Mol Pathol. (2020) 114:104435. 10.1016/j.yexmp.2020.104435 32240617

[222] Makinen LK Ahmed A Hagstrom J Lehtonen S Makitie AA Salo T. Toll-like receptors 2, 4, and 9 in primary, metastasized, and recurrent oral tongue squamous cell carcinomas. J Oral Pathol Med. (2016) 45:338–45. 10.1111/jop.12373 26426362

[223] Ren WH Zhang LM Liu HQ Gao L Chen C Qiang C. Protein overexpression of CIRP and TLR4 in oral squamous cell carcinoma: an immunohistochemical and clinical correlation analysis. Med Oncol. (2014) 31:120. 10.1007/s12032-014-0120-7 25027624

[224] Hasnat S Hujanen R Nwaru BI Salo T Salem A. The prognostic value of toll-like receptors in head and neck squamous cell carcinoma: a systematic review and meta-analysis. Int J Mol Sci. (2020) 21:7255. 10.3390/ijms21197255 33008143 PMC7582583

[225] West AC Tang K Tye H Yu L Deng N Najdovska M. Identification of a TLR2-regulated gene signature associated with tumor cell growth in gastric cancer. Oncogene. (2017) 36:5134–44. 10.1038/onc.2017.121 28481875

[226] Liu YD Yu L Ying L Balic J Gao H Deng NT. Toll-like receptor 2 regulates metabolic reprogramming in gastric cancer via superoxide dismutase 2. Int J Cancer. (2019) 144:3056–69. 10.1002/ijc.32060 30536754 PMC6590666

[227] Chefetz I Alvero AB Holmberg JC Lebowitz N Craveiro V Yang-Hartwich Y. TLR2 enhances ovarian cancer stem cell self-renewal and promotes tumor repair and recurrence. Cell Cycle. (2013) 12:511–21. 10.4161/cc.23406 23324344 PMC3587452

[228] Li D Wang X Wu JL Quan WQ Ma L Yang F. Tumor-produced versican V1 enhances hCAP18/LL-37 expression in macrophages through activation of TLR2 and vitamin D3 signaling to promote ovarian cancer progression *in vitro*. PLoS ONE. (2013) 8:e56616. 10.1371/journal.pone.0056616 23424670 PMC3570526

[229] Tye H Kennedy CL Najdovska M McLeod L McCormack W Hughes N. STAT3-driven upregulation of TLR2 promotes gastric tumorigenesis independent of tumor inflammation. Cancer Cell. (2012) 22:466–78. 10.1016/j.ccr.2012.08.010 23079657

[230] Galoczova M Coates P Vojtesek B. STAT3, stem cells, cancer stem cells and p63. Cell Mol Biol Lett. (2018) 23:12. 10.1186/s11658-018-0078-0 29588647 PMC5863838

[231] Echizen K Hirose O Maeda Y Oshima M. Inflammation in gastric cancer: interplay of the COX-2/prostaglandin E2 and toll-like receptor/MyD88 pathways. Cancer Sci. (2016) 107:391–7. 10.1111/cas.12901 27079437 PMC4832872

[232] Muhammad N Bhattacharya S Steele R Phillips N Ray RB. Involvement of c-Fos in the promotion of cancer stem-like cell properties in head and neck squamous cell carcinoma. Clin Cancer Res. (2017) 23:3120–8. 10.1158/1078-0432.CCR-16-2811 27965308 PMC5468504

